# Identification of sources of DIF using covariates in patient-reported outcome measures: a simulation study comparing two approaches based on Rasch family models

**DOI:** 10.3389/fpsyg.2023.1191107

**Published:** 2023-08-10

**Authors:** Yseulys Dubuy, Jean-Benoit Hardouin, Myriam Blanchin, Véronique Sébille

**Affiliations:** ^1^UMR INSERM 1246, MethodS in Patients-centered outcomes and HEalth ResEarch (SPHERE), Nantes Université, Nantes, France; ^2^Methodology and Biostatistics Unit, CHU Nantes, Nantes Université, Nantes, France; ^3^Public Health Department, CHU Nantes, Nantes Université, Nantes, France

**Keywords:** differential item functioning (DIF), measurement invariance (MI), Rasch measurement theory (RMT), regularization, LASSO, patient-reported outcome (PRO)

## Abstract

When analyzing patient-reported outcome (PRO) data, sources of differential item functioning (DIF) can be multiple and there may be more than one covariate of interest. Hence, it could be of great interest to disentangle their effects. Yet, in the literature on PRO measures, there are many studies where DIF detection is applied separately and independently for each covariate under examination. With such an approach, the covariates under investigation are not introduced together in the analysis, preventing from simultaneously studying their potential DIF effects on the questionnaire items. One issue, among others, is that it may lead to the detection of false-positive effects when covariates are correlated. To overcome this issue, we developed two new algorithms (namely ROSALI-DIF FORWARD and ROSALI-DIF BACKWARD). Our aim was to obtain an iterative item-by-item DIF detection method based on Rasch family models that enable to adjust group comparisons for DIF in presence of two binary covariates. Both algorithms were evaluated through a simulation study under various conditions aiming to be representative of health research contexts. The performance of the algorithms was assessed using: (i) the rates of false and correct detection of DIF, (ii) the DIF size and form recovery, and (iii) the bias in the latent variable level estimation. We compared the performance of the ROSALI-DIF algorithms to the one of another approach based on likelihood penalization. For both algorithms, the rate of false detection of DIF was close to 5%. The DIF size and form influenced the rates of correct detection of DIF. Rates of correct detection was higher with increasing DIF size. Besides, the algorithm fairly identified homogeneous differences in the item threshold parameters, but had more difficulties identifying non-homogeneous differences. Over all, the ROSALI-DIF algorithms performed better than the penalized likelihood approach. Integrating several covariates during the DIF detection process may allow a better assessment and understanding of DIF. This study provides valuable insights regarding the performance of different approaches that could be undertaken to fulfill this aim.

## 1. Introduction

Patient-reported outcome (PRO) measures have gained interest in health research to take into account patients’ perspectives on healthcare (Basch, 2017). PRO measures are often obtained via questionnaires completed by patients. These questionnaires include several items usually grouped into one or several domains to measure unobservable constructs (i.e., *latent variables*) such as fatigue or anxiety. Studies involving PRO measures often aim to compare patient levels on a latent variable by means of group comparisons and/or to study change in the latent variable. To make valid comparisons, one must ensure that individuals with different characteristics interpret the items in the same way and/or that their perception of the items remains the same over time (Sawatzky et al., 2017). However, patients’ characteristics may interfere with how some items are perceived. This phenomenon is known as *differential item functioning* (DIF). DIF occurs when patients do not interpret items in the same way according to their group membership and thus have differing item endorsement probabilities despite having the same latent variable level. In case of DIF, there is a violation of the assumption of between-group measurement invariance (Mellenbergh, 1989; Millsap and Everson, 1993; Millsap, 2011). Ignoring this lack of measurement invariance may lead to measurement bias, as observed between-group differences may not only reflect differences in the targeted latent variable (Rouquette et al., 2016). Changes in the meaning of the subjective evaluation of the target construct may also occur over time, leading to noncomparable data between time points due to a lack of longitudinal measurement invariance. This phenomenon has been acknowledged as *response shift* (Sprangers and Schwartz, 1999; Vanier et al., 2021).

There is a wide range of DIF detection methods in the literature. Among them, we can mention the Mantel-Haenszel method (Holland and Thayer, 1988), the logistic regression procedure (Rogers and Swaminathan, 1993), the likelihood-ratio test (Thissen et al., 1986, 1988, 1993) and the Lord’s chi-square (Wald) test (Lord, 2008). In the literature on PRO measures, there are many studies where DIF detection is applied separately and independently for each covariate under examination (i.e., the analysis is performed one covariate at a time). With such an approach, the covariates under investigation are not introduced together in the analysis, preventing from simultaneously studying their potential DIF effects on the questionnaire items. Yet, sources of DIF can be multiple (Zumbo, 2007; Zumbo et al., 2015; Jones, 2019); there may be more than one covariate of interest, and it may be of great interest to disentangle their effects. For instance, perception of items might differ according to gender but also age or health status. Moreover, there may be situations where two correlated covariates are investigated for DIF, but only one is really inducing DIF. In such cases, the often-encountered approach of performing the analysis separately, i.e., one covariate at a time, could lead to inferring DIF for the wrong covariate in addition to the true DIF inducing covariate, due to the correlation between the two. Employing such an approach may thus not be appropriate to disentangle DIF effects between several covariates. Therefore, more elaborated modeling strategies, allowing researchers to consider simultaneously several potentially correlated covariates when searching for DIF, could be of great interest to get more insight into the sources of measurement non-invariance.

MIMIC-model methods for DIF detection are very popular in the literature for this purpose (Woods, 2009). This approach is flexible as it can be parameterized either as: (i) a structural equation model (assuming linear relationships between the item responses and the latent variable level) or (ii) a probabilistic model from item response or Rasch measurement theory (assuming nonlinear relationships). Theoretically, MIMIC-based analyses enable the detection of DIF considering simultaneously several covariates (and their possible interaction) through the introduction of the covariates’ effects on the latent variable mean and on the item parameters (Woods, 2009; Chun et al., 2016). DIF effects are then assessed by statistical testing. Despite their popularity, the performance of MIMIC methods has been seldom evaluated. Indeed, Chun et al. (2016) indicated that no published simulation studies examined the performance of the MIMIC approaches for DIF detection when investigating two or more grouping variables and their interaction. Hence, these authors performed a simulation study to assess the DIF detection performance of three different MIMIC-based analyses: (i) the constrained baseline implementation (assumes that all items other than the one under investigation for DIF are invariant), (ii) the free baseline implementation (uses a DIF-free item assumed to be invariant and designated *a priori*) and (iii) the sequential-free baseline (uses a DIF-free item assumed to be invariant and designated based on the constrained baseline approach). In their simulations, two binary covariates and their interaction could induce DIF and were under investigation. While MIMIC methods (free-baseline and sequential-free baseline implementations) appeared to be efficient for detecting DIF items, the identification of the covariates inducing DIF seemed problematic (Chun et al., 2016).

Within the item response theory (IRT) or Rasch measurement theory (RMT) frameworks, statistical approaches have also been recently developed to consider several covariates simultaneously. On the one hand, we can mention iterative detection methods such as the IRT with covariates (IRT-C) procedure (Tay et al., 2013, 2016) and the recursive partitioning approaches [namely the partial credit model (PCM) tree, PCM-tree (El-Komboz et al., 2018) and the item-focused tree algorithm, PCM-IFT (Bollmann et al., 2018)]. Yet, these methods show some limitations. Indeed, the IRT-C procedure is only designed for dichotomous items, and the indices on which the procedure relies have been questioned (Oberski et al., 2013). Besides, the PCM-tree approach makes it hard to identify which item is affected by DIF (Bollmann et al., 2018) and the current implemented version of the PCM-IFT algorithm does not seem to model the covariates’ effect on the latent variable level (adjusted for DIF when appropriate). On the other hand, Schauberger and Mair (2020) proposed two methods based on penalized estimation of IRT or RMT models: one that only searches for a specific form of DIF having the same effects across all response categories (evaluated by simulations) and one that searches for more general forms of DIF not assuming that DIF has the same effect across all response categories (not evaluated by simulations). Data on the DIF detection performance of these penalization-based approaches in case of simultaneous covariates are lacking as simulations pertained to a specific form of DIF in polytomous items. In addition, simulated tests were always composed of 20 items, which is rarely the case in health research, where the domains of the most commonly used scales include between 2 and 10 items [e.g., SF-36, HADS or PROMIS-29 (Zigmond and Snaith, 1983; Ware and Sherbourne, 1992; Hinchcliff et al., 2011)].

In a broader issue of measurement invariance assessment, the ROSALI algorithm (Blanchin et al., 2020, 2022; Hammas et al., 2020) has been proposed in the RMT framework to detect and adjust for DIF and response shift in the analysis of longitudinal PRO data (polytomous and dichotomous items) in order to ensure valid comparisons between groups and over time. Of note, RMT was chosen to develop the ROSALI algorithm because Rasch family models possess the specific objectivity property that can be valuable when some items are missing (Blanchin et al., 2020). ROSALI is an iterative item-by-item detection algorithm that currently enables the introduction of one binary covariate in the analysis. It consists of two main parts that allow to:

- Identify items that function differently between the two groups defined by the covariate at the first measurement occasion (first part of ROSALI).
- Determine whether the perception of some items changes between two time points and assess whether or not these changes over time are similar in both groups (second part of ROSALI).

Of note, ROSALI ends by a final model allowing to adjust latent variable levels comparisons for the lack of invariance previously evidenced, if appropriate. Simulations showed that ROSALI does not erroneously infer DIF when DIF has not been simulated (Blanchin et al., 2022) and its performance to detect DIF are currently being assessed with one covariate in another study. To date, there is a will to extend ROSALI to simultaneously consider several sources of lack of invariance (e.g., gender, country). Thus, the first part of ROSALI needs to be extended to detect and adjust for DIF at one time point in presence of several covariates. However, it is currently unclear whether item-by-item iterative processes are the best approach or if it would be better to use a penalization approach that allows searching for DIF in all items simultaneously.

The aim of this study is twofold:

(1) To extend the first part of ROSALI (dedicated to the detection of DIF at one time point) to enable the simultaneous introduction of two binary covariates,
(2) To compare by simulations the detection performance of this extension to the one obtained with the approach using likelihood penalization under various conditions, including moderate numbers of polytomous items (representative of PRO instrument subscales used in health research), moderate sample sizes, potentially correlated covariates, and various forms of DIF.

## 2. Materials and methods

### 2.1. Rasch measurement theory

Rasch measurement theory is a family of models derived from the Rasch model for dichotomous items (Fischer and Molenaar, 1995). For polytomous items, the most flexible model is the PCM (Masters, 1982; Fischer and Ponocny, 1994), its formulation for a questionnaire composed of *J* polytomous items with *M*_*j*_ response categories for item *j* (*j* = 1, …, *J*) is given by:


$$\mathbb{P}\left(X_{i\,j}=x|\theta_{i},\delta_{j\,1},\ldots ,\delta_{j\,M_{j}-1}\right)=\frac{\text{exp}\,\left(x\,\theta_{i}-\sum_{p=1}^{x}\delta_{j\,p}\right)}{\sum_{l=0}^{M_{j}-1}\exp\left(l\,\theta_{i}-\sum_{p=1}^{l}\delta_{j\,p}\right)}$$

**FIGURE 1:**
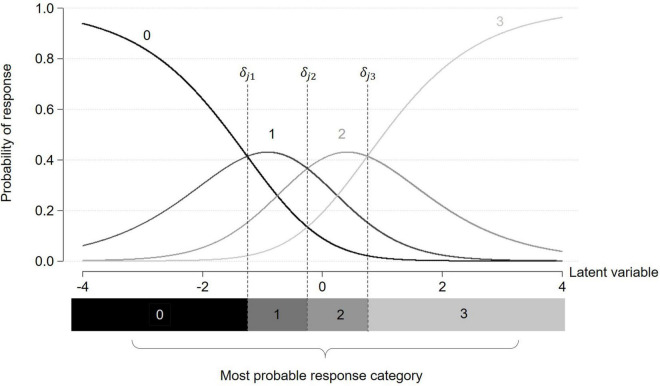
Category characteristic curves for a given item *j* with four response categories under a partial credit model. Item threshold parameters δ_*jp*_ are indicated by dashed lines.

The conditional probability that an individual *i* answers *x* (= 0, 1, …, *M*_*j*_ − 1) to item *j* is a function of:

- The latent variable level of individual *i*: θ_*i*_
Where θ_*i*_ is the realization of Θ, a random variable assumed normally distributed (with mean μ and standard deviation σ). This latent variable is assumed to represent the target construct (e.g., anxiety).
- The *item threshold parameters* δ_*jp*_ associated with each response category *p* > 0 of item *j* (1 ≤ *p* ≤ *M*_*j*_ − 1). δ_*jp*_ represents the latent variable level at which the probabilities of answering category *p* or *p-1* to item *j* are equal. When tracing the probability curves of each response category, item threshold parameters (e.g., δ_*j*1_) correspond to the intersection between two adjacent category probability curves as pictured in Figure 1 (Christensen et al., 2012).

### 2.2. DIF in RMT

**FIGURE 2:**
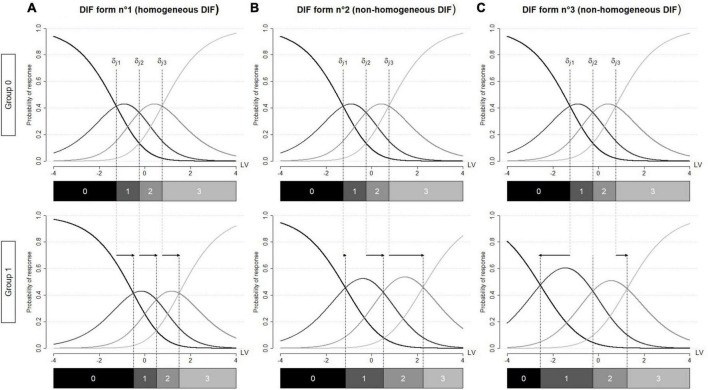
Category characteristic curves for a given item j affected by differential item functioning (DIF). DIF is operationalized by between-group differences in the item threshold parameters. These differences are represented by arrows. Graph **(A)** between-group differences in the item threshold parameters have the same direction and the same magnitude (homogeneous DIF). Graph **(B)** between-group differences in the item threshold parameters have the same direction but vary in magnitude (non-homogeneous DIF) Graph **(C)** between-group differences in the item threshold parameters vary in direction and magnitude (non-homogeneous DIF). LV, latent variable.

Rasch family models are often used to detect DIF in health research (Rouquette et al., 2019). Within RMT, DIF has been operationalized as between-group differences in the item threshold parameters. Dichotomous items are characterized by a single threshold parameter. Hence, DIF in dichotomous items can only take on a single form, where category probability curves are shifted between groups [i.e., parallel uniform DIF (Millsap, 2011)]. However, polytomous items are characterized by several threshold parameters (one for each response category above 0). Therefore, between-group differences in the item threshold parameters may vary in magnitude, direction, or both, leading to numerous potential DIF forms (Penfield, 2007). For instance, between-group differences in the item threshold parameters can:

(1) Have the same direction and the same magnitude, e.g., Figure 2A.

(2) Have the same direction but vary in magnitude, e.g., Figure 2B.
(3) Vary in direction and magnitude, e.g., Figure 2C.

In the manuscript, the forms described in (2) and (3) will be referred to as *non-homogeneous DIF* (Bollmann et al., 2018). Of note, these two forms illustrate respectively the *convergent* and *divergent differential step functioning* introduced by Penfield (2007) and Penfield et al. (2009). To maintain a consistent terminology throughout the manuscript, the form described in (1) will be referred to as *homogeneous DIF*. Of note, Penfield et al. (2009) referred to it to as *pervasive constant differential step functioning* and many researchers use the term DIF [e.g., (Bollmann et al., 2018; Schauberger and Mair, 2020)].

The PCM can be used to assess the impact of a binary covariate *C* on the latent variable level accounting for a potential DIF induced by *C* through the introduction of group effects on the latent variable level and on the item threshold parameters:


$$\begin{array}{c} \mathbb{P}\left(X_{i\,j}=x|\theta_{i},C_{i},\beta ,\delta_{j\,1},\ldots ,\delta_{j\,M_{j}-1},\gamma_{j\,1},\ldots ,\gamma_{j\,M_{j}-1}\right)=\\ \frac{\text{exp}\left(x\left[\theta_{i}+\beta .C_{i}\right]-\sum_{p=1}^{x}\left[\delta_{j\,p}+\gamma_{j\,p}.C_{i}\right]\right)}{\sum_{l=0}^{M_{j}-1}\exp\left(l\left[\theta_{i}+\beta .C_{i}\right]-\sum_{p=1}^{l}\left[\delta_{j\,p}+\gamma_{j\,p}.C_{i}\right]\right)}\;\left(1\right) \end{array}$$

In addition to the above-mentioned parameters, we have:

- *C*_i_ the realization of covariate *C* for individual *i*. *C*_i_ equals either 0 (reference group) or 1.

- β the effect of covariate *C* on the latent variable level (sometimes referred to as the *group effect*). β equals the difference between μ_1_ and μ_0_, where μ_1_ designates the latent variable mean in the group of individuals with *C*_*i*_ = 1, and μ_0_ designates the latent variable mean in the group *C*_*i*_ = 0 (β = μ_1_−μ_0_).

- γ_*jp*_ the DIF parameters interfering with the item thresholds and modeling the DIF effects of covariate *C*. These DIF parameters operationalize the difference in item threshold parameters between the groups. Item threshold parameters in the reference group are δ_*jp*_ and item threshold parameters in the focal group are equal to δ_*jp*_ + γ_*jp*_. If there is no DIF on item *j*, then γ_*jp*_ = 0.

Additional binary covariates can be added in the same way. For instance, with two covariates C_1_ and C_2_, without interaction:


(2) ℙ(Xi⁢j=x|θi,C1i,β1,C2i,β2,δj⁢1,…,δj⁢Mj-1,γj⁢1(C1),…,γj⁢Mj-1(C1),

$$\gamma_{j\,1}^{\left(C_{2}\right)},\ldots ,\gamma_{j\,M_{j}-1}^{\left(C_{2}\right)})=$$

exp(x[θi+β1.C1i+β2.C2i]-∑p=1x[δj⁢p+γj⁢p(C1).C1i+γj⁢p(C2).C2i])∑l=0Mj-1exp(l[θi+β1.C1i+β2.C2i]-∑p=1l[δj⁢p+γj⁢p(C1).C1i+γj⁢p(C2).C2i])

### 2.3. DIF detection procedures

#### 2.3.1. Extension of the first part of ROSALI

The first part of the ROSALI algorithm with one binary covariate has been described elsewhere (Hammas et al., 2020; Blanchin et al., 2022). We extended this algorithm by adding a second binary covariate. DIF detection then relies on the following steps:

**Step 1.** Estimation of a fully non-invariant PCM where the two covariates are assumed to induce DIF on all items.
**Step 2.** Estimation of a fully invariant PCM (no DIF is assumed).
**Step 3.** Test of the global occurrence of DIF by comparing the two previous models using a likelihood-ratio test (LRT).
**Step 4.** If the LRT is significant, screen all item-covariate pairs for DIF separately based on the fully non-invariant model. Otherwise, go to step 6.
**Step 5.** Forward iterative selection of the significant DIF item-covariate pairs found in step 4 (starting from the fully invariant model) and assessment of the form of DIF involved. A Bonferroni correction is performed to account for multiple testing.
**Step 6.** Estimation of a final model giving the covariates effect on the latent variable level adjusted for DIF (if appropriate).

**FIGURE 3:**
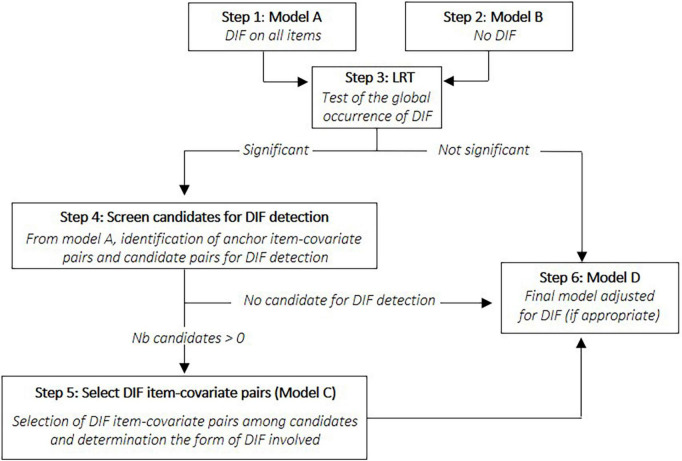
Graphical representation of the two ROSALI-DIF algorithms (ROSALI-DIF FORWARD and ROSALI-DIF BACKWARD). DIF, differential item functioning; LRT, likelihood-ratio test; Nb, number.

This extension of the first part of ROSALI will be referred to as ROSALI-DIF FORWARD. All steps are comprehensively described in Table 1 alongside statistical considerations. An alternative version of this algorithm has also been explored, with the same philosophy, but with an iterative step based on a backward instead of a forward process where all candidate pairs are tested simultaneously instead of one-by-one. This alternative version has been named ROSALI-DIF BACKWARD and is described in Supplementary Appendix A. Both algorithms are jointly pictured in Figure 3. Of note, these algorithms were designed to be easily extendable to the situation where more than two covariates are under investigation, or when continuous covariates are considered instead of binary covariates. Both algorithms can be seen as an iterative MIMIC approach for DIF detection. However, to date, they do not enable to consider the interaction between the covariates. Of note, the screening step (step 4) was inspired by the iterative Wald test procedure (Tay et al., 2015; Cao et al., 2017).

**TABLE 1:** Comprehensive description of the ROSALI-DIF FORWARD algorithm and statistical considerations.

ROSALI-DIF FORWARD steps	Statistical considerations
**Step 1: Estimation of a fully non-invariant model (Model A)** A fully unconstrained PCM is estimated in this first step where the two binary covariates *C*_1_ and *C*_2_ are assumed to induce DIF on all items	All DIF parameters $\gamma_{j\,p}^{\left(C_{1}\right)}$ and $\gamma_{j\,p}^{\left(C_{2}\right)}$ are freely estimated (∀*j* and *p*) in Equation 2. **Identifiability constraints:** the effects of covariates on the latent variable level are constrained to 0 (β_1_ = β_2_ = 0).
**Step 2: Estimation of a fully invariant model (Model B)** A fully constrained model assuming no DIF is estimated in this second step.	All DIF parameters $\gamma_{j\,p}^{\left(C_{1}\right)}$ and $\gamma_{j\,p}^{\left(C_{2}\right)}$ are constrained to zero (∀*j* and *p*) in Equation 2. The effects of covariates on the latent variable level (i.e., β_1_ and β_2_) are freely estimated.
**Step 3. Test of the global occurrence of DIF** The third step aims to evaluate the global occurrence of DIF by comparing model A and model B using a likelihood-ratio test. If the test is not significant, we assume that the covariates do not induce DIF and the algorithm moves directly to step 6 where the final model is model B. Otherwise, we proceed to the next step.	**Rationale for the likelihood-ratio test:** Model B is nested in Model A **Significance level:** 5%
**Step 4. Screen item-covariate pairs (Item j**, **Covariate C**) **candidate for DIF detection** From Model A (where the two covariates induce DIF on all items), statistical tests are performed for each item-covariate pair separately to determine whether the DIF effect induced by covariate *C* on item *j* is significant or not. Candidate pairs are those associated with significant tests. Measurement invariance is assumed for the other pairs (anchor pairs). Of note, if no pairs are considered as candidate, the algorithm goes directly to step 6 where the final model is model B.	**Statistical tests:** Contrast tests **Null and alternative hypotheses of contrast test for DIF:** H_0_)$\forall p,\gamma_{j\,p}^{\left(C\right)}$ = 0 (No DIF) H_1_) $\exists p:\gamma_{j\,p}^{\left(C\right)}$ ≠ 0 (DIF) **Significance level:** 5%
**Step 5. Selection of DIF item-covariate pairs (Item j**, **Covariate C**) **among candidate pairs and assessment of the form of DIF involved** This step is an iterative step that aims to select the item-covariate pairs affected by DIF among candidate pairs and determine the form of DIF involved. A new model (Model C) is introduced so that Model C = Model B at the beginning of this step. From model C, we estimate new models (one for each candidate pair) where the invariance constraint associated with the pair of interest is relaxed, and other constraints remain unchanged. From these new models, statistical tests are performed for each pair to determine whether the DIF effect induced by covariate *C* on item *j* is significant or not. We retain the model with the pair having the most significant test (smallest *p*-value) after Bonferroni correction. The associated pair is assumed to be affected by DIF and will be denoted (item j*, covariate C*). If there is no significant pair, the algorithm moves to step 6. Otherwise, based on the retained model, the form of DIF induced by covariate C* on item j* is assessed using another test. Model C is updated to account for the evidenced DIF and its form. The retained pair will no longer be tested. Step 5 is repeated over the remaining pairs to be tested. The step ends if no more pair is retained, if all candidate pairs have been tested, or just before relaxing the invariance constraint of the last anchor item for a given covariate.	********** Test DIF effect of candidate pairs ********** **Null and alternative hypotheses of contrast test for DIF**: H_0_)$\forall p,\gamma_{j\,p}^{\left(C\right)}$ = 0 (No DIF) H_1_) $\exists p:\gamma_{j\,p}^{\left(C\right)}$ ≠ 0 (DIF) **Significance level:** 5%/number of candidate pairs, Bonferroni correction performed to avoid the inflation of the type I error rate due to multiple testing. ********** Test DIF form on the retained pair ********** **Null and alternative hypotheses of contrast test to assess DIF form:** H_0_)$\forall p,\gamma_{j^{*}\,p}^{\left(C^{*}\right)}$ = $\gamma_{j^{*}}^{\left(C^{*}\right)}$ (Homogeneous DIF) H_1_)∃*p*,*p*′: $\gamma_{j^{\text{*}}p}^{\left(C^{\text{*}}\right)}\neq \gamma_{j^{\text{*}}p^{\text{'}}}^{\left(C^{\text{*}}\right)}$ (Non-homogeneous DIF) **Significance level:** 5% *************** Update Model C *************** If the previous test is significant, the DIF parameters $\gamma_{j^{*}\,p}^{\left(C^{*}\right)}$ associated with the retained pair are freely estimated (non-homogeneous DIF). Otherwise, the DIF parameters $\gamma_{j^{*}\,p}^{\left(C^{*}\right)}$ are estimated but constrained to be constant over all response categories (homogeneous DIF).
**Step 6. Estimation of the covariates effect on the latent variable level (Model D)** The last step estimates the effect of the covariates *C*_*1*_ and *C*_*2*_ on the latent variable level adjusted for the DIF that was previously evidenced, if appropriate, using a final model called model D.	Model D = Model B if no DIF has been evidenced. Otherwise, model D is equal to the last version of model C obtained at the end of step 5.

C designates here indistinctly covariates C_1_ or C_2_. This algorithm estimates several PCMs derived from Equation 2 with marginal maximum likelihood estimation. For all PCMs, the variances of the latent variable distribution across groups are assumed equal.

#### 2.3.2. Likelihood penalization approach

A DIF detection method for polytomous items using likelihood penalization of a PCM or a generalized PCM (GPCM) has been comprehensively described by Schauberger and Mair (2020). By using likelihood penalization, the authors translated DIF detection into a parameter selection problem and aimed to determine which DIF parameters $\gamma_{j\,p}^{\left(C\right)}$ are worth estimating. The number of DIF parameters to be estimated depends entirely on the choice of the tuning parameter which controls the strength of the penalization. When this parameter is equal to zero, all DIF parameters are estimated (no penalization). On the contrary, no DIF parameters are estimated when this parameter tends to +∞.

**FIGURE 4:**
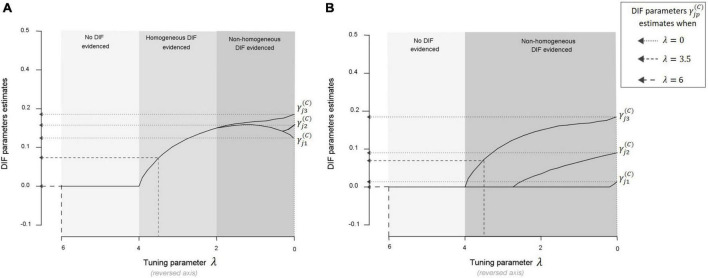
Graph **(A)** DIF parameters $\gamma_{j\,p}^{\left(C\right)}$ paths with the PCMLasso approach for a given item-covariate pair in a fictious example (configuration A: the graph shows an area where the DIF parameters $\gamma_{j\,p}^{\left(C\right)}$ from Equation 2 are estimated but constrained to be equal). When the tuning parameter is large (left of the graph: λ ∈ [6, 4]), no DIF parameter is estimated since the penalization is strong. In the middle of the graph (λ ∈ [4, 2]), the penalization is not as strong, allowing the estimation of all DIF parameters (constrained to be equal over the response categories). Finally, at the right of the graph (λ ∈ [2, 0]), the penalization is weak and the DIF parameters are no longer constrained to be equal over the response categories. If the optimal tuning parameter λ falls in the area [6, 4], then no DIF is evidenced for the item-covariate pair considered. If it falls in the area [4, 2] (respectively [2, 0]), then homogeneous (respectively non-homogeneous) DIF is evidenced for the given pair. Graph **(B)** DIF parameters paths with the PCMLasso approach for a given item-covariate pair (in this configuration B, the graph does not show an area where the DIF parameters are estimated but constrained to be equal, i.e., it does not show an area where homogeneous DIF could be evidenced). When the tuning parameter is large (left of the graph: λ ∈ [6, 4]), no DIF parameter is estimated since the penalization is strong. Then, the penalization decreases, allowing the estimation of a first DIF parameter ($\gamma_{j\,3}^{\left(C\right)}$), then a second ($\gamma_{j\,2}^{\left(C\right)}$) and finally a third one ($\gamma_{j\,1}^{\left(C\right)}$). If the optimal tuning parameter λ falls in the area [6, 4], then no DIF is evidenced for the item-covariate pair considered, otherwise, non-homogeneous DIF is evidenced.

In practice, PCMs (or GPCMs) are estimated across a wide range of tuning parameters (one model for each value of tuning parameter). All estimates of the DIF parameters $\gamma_{j\,p}^{\left(C\right)}$ related to a given item-covariate pair are then plotted as functions of the tuning parameter values in graphs called *DIF parameters paths* (see Figure 4). The optimal tuning parameter is chosen to minimize the Bayesian information criterion. DIF is evidenced on a given item-covariate pair if and only if one of the DIF parameters related to the pair is estimated to be different from 0 (no statistical tests are performed for this procedure). Two main situations can arise when searching for DIF as pictured in Figure 4: either the graph shows an area where the DIF parameters $\gamma_{j\,p}^{\left(C\right)}$ are estimated but constrained to be equal [graph (A)], or the graph does not show such an area [graph (B)]. The form of DIF evidenced is therefore entirely determined by the choice of the tuning parameter.

We chose to evaluate this approach, named PCMLasso, enabling the detection of both forms of DIF using a PCM. Of note, the PCMLasso approach relies on a PCM where the item discrimination parameters are constrained to be equal over all items. In the ROSALI-DIF algorithms, the discrimination parameters equal 1 for all items. Besides, neither the ROSALI-DIF algorithms nor PCM-Lasso enable to consider that the DIF effect of one covariate may depend on the level of another covariate.

### 2.4. Simulation study

#### 2.4.1. Data simulation

We simulated the responses of *n* = 400 or 800 individuals to a unidimensional questionnaire composed of *J* = 4 or 7 polytomous items (item 1, …, item *J*) with *M* = 4 response categories, numbered from 0 to *M-1*. Individual latent variable levels were drawn from a standard normal distribution and responses were generated by a PCM. Item threshold parameters δ_*jp*_ were chosen to cover all the latent variable continuum (values are given in Supplementary Appendix B. Items were numbered from 1 to *J* so that $\bar{\delta_{1\,p}}<\bar{\delta_{2\,p}}<\ldots <\bar{\delta_{J\,p}}$ (where $\bar{\delta_{j\,p}}$ stands for the average value of the item *j* threshold parameters).

The structure of the questionnaire (number of items and number of response categories) was chosen to be in accordance with the unidimensional subscales of PRO instruments commonly encountered in health research; e.g., SF-36 (Ware and Sherbourne, 1992), QLQ-C30 (Aaronson et al., 1993), HADS (Zigmond and Snaith, 1983) and PROMIS-29 (Hinchcliff et al., 2011). Regarding the number of individuals, a wide range of sample sizes can be found in the literature on PRO measures. DIF can be investigated in:

(i) Studies on psychometric properties of a PRO instrument. Anthoine et al. (2014) estimated that sample sizes of such studies ranged from 24 to 7,906, with a mean sample size of 509 (standard deviation = 1094) and a median equal to 207,
(ii) Analyses of data from observational studies including PRO measures. Based on our practical experience in France, cohorts generally contain about 300 to 500 patients [e.g., PreKitQol (Sébille et al., 2016), ELCCA (Bourdon et al., 2016) and FATSEIN (Rotonda et al., 2011)], but some cohorts are also larger, notably within population-based cohort [e.g., VICAN (Bouhnik et al., 2015)],
(iii) Studies on data collected within clinical trials, where sample size generally range between 100 to 1,000, often equally distributed between the treatment arms (Glas et al., 2009; Loubert et al., 2022).

Hence, we chose to set the sample size *n* at 400 and 800 as it seemed to represent a good compromise. However, smaller sample sizes are also frequent and larger sample sizes can also be encountered.

#### 2.4.2. DIF operationalization

DIF was operationalized as between-group differences in item threshold parameters^1^ (groups being defined by observed covariates). For this simulation study, two binary covariates (denoted *C*_1_ and *C*_2_) were considered as possibly inducing DIF on items. Three settings were derived:

**Setting No. 1:** The two covariates were not correlated and they each induced DIF on a different item.

**Setting No. 2:** The two covariates were not correlated and they induced DIF on the same item.

**Setting No. 3:** The two covariates were correlated and only one (i.e., *C*_1_) induced DIF on two items. Further details on the process used to obtain correlated covariates are available in online Supplementary Appendix B alongside the formulas used for the DIF-items threshold parameters among each setting.

For each setting, two different forms of DIF were explored: homogeneous and non-homogeneous DIF. Homogeneous DIF was operationalized as between-group differences in item threshold parameters with the same direction and magnitude across the response categories (i.e., $\forall p,\gamma_{j\,p}^{\left(C\right)}=\gamma_{j}^{\left(C\right)},$Figure 2A). Non-homogeneous DIF was operationalized as between-group differences in item threshold parameters that varied in magnitude and/or direction. In our simulations, we only simulated between-group differences having the same direction but different magnitudes. Specifically, we shifted item threshold parameters by increasing values (e.g., Figure 2B). Finally, we varied the DIF size (weak or medium). Table 2 contains a comprehensive description of the magnitudes considered for the DIF parameters $\gamma_{j\,p}^{\left(C\right)}$ according to the DIF size and form. Of note, DIF sizes were chosen based on previous literature on DIF simulation (Rouquette et al., 2016; Tay et al., 2016; Bollmann et al., 2018). A comprehensive summary of the simulation study appears in Table 2. The combination of all simulation parameters led to 48 scenarios. We added 8 scenarios with no DIF as control scenarios. Each scenario was replicated 500 times and resulting datasets were then analyzed with the three DIF detection procedures (i.e., ROSALI-DIF algorithms and PCMLasso).

**TABLE 2:** Simulation plan summary.

Questionnaire and sample features	
Number of items (*J*)	*J* = 4, 7 items
Number of response categories (*M*)	*M* = 4 response categories per item
Sample size (*n*)	*n* = 400, 800 simulated individuals
**Latent variable (Θ)**	
Mean μ, variance σ^2^	μ = 0, σ^2^ = 1
Main effect of covariates on latent variable level	No main effect of covariate *C*_1_: β_1_ = 0 No main effect of covariate *C*_2_: β_2_ = 0
**DIF inducing covariates**	
Setting No. 1	Covariates *C*_*1*_ and *C*_*2*_ are uncorrelated, they each induce DIF on a different item
Setting No. 2	Covariates *C*_*1*_ and *C*_*2*_ are uncorrelated, they both induce DIF on the same item
Setting No. 3	Covariates *C*_*1*_ and *C*_*2*_ are correlated, only covariate *C*_*1*_ induces DIF on two items
**DIF items**	
***J* = *4 items***	
Setting No. 1	Covariate *C*_1_ induces DIF on item 2and covariate *C*_2_ induces DIF on item 3
Setting No. 2	Covariates *C*_1_ and *C*_2_ induce DIF on item 2
Setting No. 3	Covariate *C*_1_ induces DIF on items 2 and 3
***J* = *7 items***	
Setting No. 1	Covariate *C*_1_ induces DIF on item 3and covariate *C*_2_ induces DIF on item 5
Setting No. 2	Covariates *C*_1_ and *C*_2_ induce DIF on item 3
Setting No. 3	Covariate *C*_1_ induces DIF on items 3 and 5
**DIF form**	
Homogeneous	Same effect (magnitude and direction) of the covariate on all item threshold parameters: $\forall p,\gamma_{j\,p}^{\left(C\right)}=\gamma_{j}^{\left(C\right)}$
Non-homogeneous	The covariate has varying effects across the item threshold parameters
**DIF size**	
**Weak**	
Homogeneous DIF	$\forall p,\gamma_{j\,p}^{\left(C\right)}=\gamma_{j}^{\left(C\right)}=0.3$
Non homogeneous DIF	$\gamma_{j\,1}^{\left(C\right)}=0.1$, $\gamma_{j\,2}^{\left(C\right)}=0.3$, $\gamma_{j\,3}^{\left(C\right)}=0.5$
**Medium**	
Homogeneous DIF	$\forall p,\gamma_{j\,p}^{\left(C\right)}=\gamma_{j}^{\left(C\right)}=0.5$
Non Homogeneous DIF	$\gamma_{j\,1}^{\left(C\right)}=0.1$, $\gamma_{j\,2}^{\left(C\right)}=0.5$, $\gamma_{j\,3}^{\left(C\right)}=0.9$

*C* = *C*_1_ or *C*_2_.

#### 2.4.3. Evaluation criteria

The performance of the three procedures in terms of DIF detection were evaluated according to different criteria.

Firstly, the rate of false detection of DIF among scenarios with no simulated DIF was computed as the proportion of datasets where DIF was wrongly detected on at least one item-covariate pair at the end of the procedures. We expected this rate to be low, but with no predefined threshold. As the ROSALI-DIF algorithms involve a LRT performed at the 5% significance level, we also considered the proportion of datasets with a significant LRT to confront it to the nominal rate of 5%. A difference between the proportion of datasets with a significant LRT and the rate of false detection of DIF indicates that for some datasets, overall occurrence of DIF was initially suspected following the LRT, but finally not retained at the end of the procedure.

**FIGURE 5:**
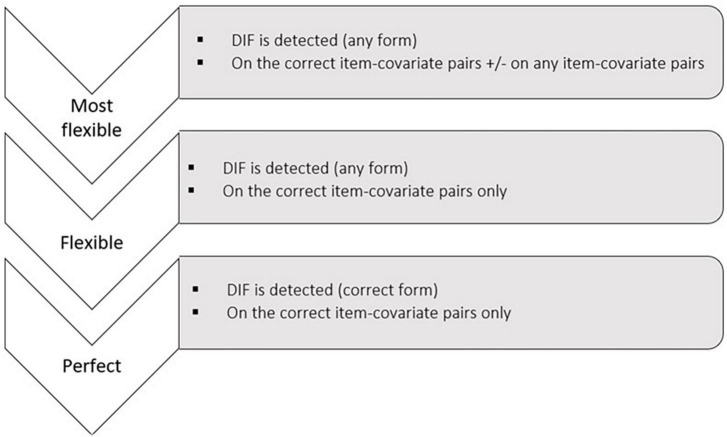
Criteria used to evaluate the DIF detection performance of the different procedures.

Secondly, among scenarios with simulated DIF, we used a set of criteria to assess the performance of the different procedures to detect DIF. They are given in Figure 5 by increasing level of requirement:

- **Most flexible criterion:** Did the procedure detect DIF on at least the correct item-covariate pairs (i.e., the pairs on which DIF was simulated)? The Most Flexible criterion is satisfied for a given dataset if the procedure correctly detects DIF on the item-covariate pairs for which it was simulated, regardless of whether other pairs (for which DIF was not simulated) are also wrongly flagged.
- **Flexible criterion:** Did the procedure detect DIF on the correct item-covariate pairs only? The Flexible criterion is met on a given dataset if the procedure only detects DIF on the item-covariate pairs for which DIF has been simulated. Hence, the criterion is not met if other item-covariate pairs are wrongly flagged.
- **Perfect criterion:** Did the procedure exactly detect what was simulated (i.e., DIF detected only on the correct item-covariate pairs and form of DIF rightly determined)? The Perfect criterion is met on a given dataset if the procedure: i) only detects DIF on the item-covariate pairs for which DIF has been simulated and ii) recovers the form of DIF simulated for each pair.

The performance of the ROSALI-DIF algorithms and PCMLasso were assessed using the proportion of datasets meeting the Most Flexible, Flexible, and Perfect criteria at the end of the procedures. Although there is no predefined threshold, high proportions of datasets meeting the different criteria indicate good performance of the different procedures.

Subsequently, we studied the difference between the proportions of datasets meeting the different criteria. For a given procedure, the proportion of datasets meeting the Most flexible criterion but not meeting the Flexible criterion indicates in what proportion the procedure has identified more item-covariate pairs affected by DIF than simulated. Similarly, the proportion of datasets meeting the Flexible criterion but not meeting the Perfect criterion indicates in which proportion the procedure detected the correct item-covariate pairs on which DIF was simulated (and only these) but failed to identify the form of DIF involved.

Finally, we assessed the bias in the estimation of the covariates’ effects on the latent variable level (β_1_ and β_2_ in Equation 2) to determine whether the three methods enable for an unbiased estimation after DIF detection. In addition to bias, we computed the standard deviation of the β_*k* (*k* = 1, 2)_ estimates and the average model standard errors. We also compared the estimates of the DIF parameters $\hat{\gamma_{j\,p}^{\left(C\right)}}$ with the true simulated values using boxplots.

Stata software release 16 was used for data generation [*simirt* module, version 4.3 (Hardouin, 2013)]. Analyses were performed using either Stata 16 for ROSALI-DIF algorithms or R 4.1.0 for the PCMLasso approach (*GPCMLasso* package version 0.1-5).

#### 2.4.4. Summary

We aimed to determine, through a simulation study, whether the ROSALI-DIF algorithms (FORWARD and BACKWARD) and PCMLasso were able to recover the DIF conditions that were simulated, that is: conclude that DIF is absent when DIF has not been simulated and identify the correct DIF item-covariate pairs when DIF has been simulated. Scenarios considered within the simulation study aimed to be representative health research contexts (quite short questionnaires, polytomous items, and moderate sample size). We explored three broad settings. The first one (setting No. 1) aimed to evaluate the procedures’ performance in a simple situation where two uncorrelated covariates induce DIF on a different item. The second setting was considered to determine whether the procedures could disentangle DIF effects when both covariates induce DIF on the same item. Finally, with setting No. 3, we determined whether the procedures could correctly identify the DIF-inducing covariate when to correlated covariates are introduced in the analysis. As it was an exploratory simulation study, we had no *a priori* on which procedure could perform better. However, we expected that performance would increase with increasing sample size and DIF size. For the ROSALI-DIF algorithms, we also expected that we might lack power to identify non-constant shifts in item thresholds (i.e., non-homogeneous DIF) as previous issues were found in a longitudinal framework (Blanchin et al., 2020, 2022).

## 3. Results

### 3.1. Rates of false DIF detection – no DIF scenarios

Table 3 presents the rates of false DIF detection for both ROSALI-DIF FORWARD and PCMLasso. It additionally gives the proportion of datasets with a significant LRT for ROSALI-DIF FORWARD.

**TABLE 3:** Rates of false detection of DIF among scenarios with no simulated DIF computed at the end of each procedure (%DIF wrongly detected) and rates of significant likelihood-ratio tests (%LRT SIG).

			ROSALI-DIF FORWARD		PCMLasso
**n**	***J***	**Corr**	**%LRT SIG**	**%DIF wrongly detected**	**%DIF wrongly detected**
400	4	No	5%	4%	50%
400	4	Yes	6%	3%	50%
400	7	No	7%	6%	44%
400	7	Yes	6%	3%	54%
800	4	No	6%	4%	46%
800	4	Yes	5%	3%	47%
800	7	No	6%	5%	48%
800	7	Yes	4%	3%	46%

%DIF wrongly detected: proportion of datasets where DIF was wrongly detected on at least one item-covariate pair at the end of the procedure (i.e., rate of false DIF detection). %LRT SIG: Proportion of datasets with a significant likelihood-ratio test. Corr: correlation, indicates whether covariates *C*_1_ and *C*_2_ are correlated (=Yes) or not (=No). The procedures converged on all datasets. No identifiability issues were encountered. Results are given according to the simulation characteristics *n* (sample size), *J* (number of items) and the presence or absence of correlation between covariates *C*_1_ and *C*_2_.

The proportions of datasets where DIF was wrongly detected on at least one item-covariate pair at the end of the algorithm were low for ROSALI-DIF FORWARD (from 3 to 6%). Neither the sample size *n* nor the number of items *J* seemed to impact these rates. However, rates of false detection of DIF were usually slightly lower when the two covariates *C*_*1*_ and *C*_*2*_ were correlated than when they weren’t. For each scenario, the rate of false detection of DIF was systematically lower than the rate of datasets with a significant LRT. It means that for some datasets (1–3%), overall occurrence of DIF was initially suspected following the LRT, but finally not retained at the end of the algorithm. Of note, the proportion of datasets with a significant likelihood-ratio test (LRT) was close to 5% (the significance level used for this test). Same results were obtained for ROSALI-DIF BACKWARD (Supplementary Appendix C).

DIF was wrongly detected on at least one item-covariate pair at the end of the PCMLasso in almost half of the datasets whatever the scenario. None of the simulation characteristics seemed to have an impact on these results. As the number of item-covariate pairs incorrectly flagged for DIF by PCMLasso among the “No DIF scenarios” remained small (one item-covariate pair on average among replications where DIF was wrongly inferred), we chose to continue the investigations in order to draw a comprehensive view of its performance (despite the high rates of false DIF detection).

### 3.2. Rates of correct DIF detection – DIF scenarios

Table 4 presents the proportion of datasets meeting the Most Flexible, Flexible and Perfect criteria at the end of the procedures for ROSALI-DIF FORWARD and PCMLasso. Results for ROSALI-DIF BACKWARD appear in Supplementary Appendix C.

**TABLE 4:** Rates of correct DIF detection among DIF scenarios.

Setting	DIF form	DIF size	n	J	ROSALI-DIF FORWARD				PCMLasso		
					**%LRT SIG**	**Most flexible**	**Flexible**	**Perfect**	**Most flexible**	**Flexible**	**Perfect**
1	H	Weak	400	4	31%	4%	3%	2%	5%	3%	0%
1	H	Weak	400	7	27%	5%	4%	3%	6%	3%	0%
1	H	Weak	800	4	64%	20%	17%	14%	16%	9%	1%
1	H	Weak	800	7	63%	26%	18%	14%	18%	9%	0%
1	H	Medium	400	4	85%	39%	34%	29%	35%	17%	0%
1	H	Medium	400	7	79%	44%	30%	25%	39%	16%	0%
1	H	Medium	800	4	99%	90%	73%	67%	80%	42%	1%
1	H	Medium	800	7	99%	91%	66%	59%	83%	46%	2%
1	NH	Weak	400	4	33%	5%	4%	1%	8%	6%	5%
1	NH	Weak	400	7	31%	4%	2%	0%	6%	3%	3%
1	NH	Weak	800	4	68%	29%	25%	3%	27%	18%	17%
1	NH	Weak	800	7	62%	27%	18%	2%	23%	14%	12%
1	NH	Medium	400	4	91%	56%	49%	6%	49%	26%	25%
1	NH	Medium	400	7	79%	51%	35%	5%	49%	24%	23%
1	NH	Medium	800	4	100%	96%	77%	31%	88%	49%	49%
1	NH	Medium	800	7	100%	96%	65%	24%	90%	52%	51%
2	H	Weak	400	4	29%	3%	1%	1%	5%	3%	0%
2	H	Weak	400	7	26%	2%	2%	1%	6%	2%	0%
2	H	Weak	800	4	68%	23%	20%	18%	17%	9%	0%
2	H	Weak	800	7	58%	21%	13%	10%	17%	8%	0%
2	H	Medium	400	4	83%	41%	35%	30%	35%	15%	0%
2	H	Medium	400	7	82%	41%	30%	25%	43%	15%	1%
2	H	Medium	800	4	99%	91%	77%	68%	76%	37%	1%
2	H	Medium	800	7	99%	92%	65%	61%	85%	46%	2%
2	NH	Weak	400	4	34%	6%	6%	1%	10%	6%	6%
2	NH	Weak	400	7	32%	7%	5%	1%	9%	5%	4%
2	NH	Weak	800	4	74%	34%	32%	2%	26%	16%	15%
2	NH	Weak	800	7	67%	32%	22%	2%	27%	15%	13%
2	NH	Medium	400	4	86%	50%	42%	5%	47%	27%	26%
2	NH	Medium	400	7	79%	51%	39%	6%	46%	22%	22%
2	NH	Medium	800	4	100%	95%	81%	26%	86%	54%	53%
2	NH	Medium	800	7	99%	96%	65%	22%	89%	51%	50%
3	H	Weak	400	4	21%	2%	2%	1%	2%	0%	0%
3	H	Weak	400	7	23%	2%	2%	2%	1%	1%	0%
3	H	Weak	800	4	44%	7%	7%	6%	2%	0%	0%
3	H	Weak	800	7	44%	13%	10%	8%	9%	3%	0%
3	H	Medium	400	4	58%	19%	18%	13%	7%	2%	0%
3	H	Medium	400	7	67%	26%	22%	18%	20%	6%	0%
3	H	Medium	800	4	91%	69%	61%	54%	19%	3%	0%
3	H	Medium	800	7	96%	78%	62%	56%	60%	21%	1%
3	NH	Weak	400	4	24%	3%	2%	0%	3%	1%	1%
3	NH	Weak	400	7	28%	2%	1%	0%	4%	1%	1%
3	NH	Weak	800	4	48%	13%	11%	1%	8%	4%	4%
3	NH	Weak	800	7	52%	17%	13%	2%	15%	6%	6%
3	NH	Medium	400	4	73%	31%	28%	5%	22%	8%	8%
3	NH	Medium	400	7	75%	38%	31%	6%	34%	16%	16%
3	NH	Medium	800	4	98%	86%	73%	25%	58%	19%	19%
3	NH	Medium	800	7	98%	85%	66%	26%	78%	34%	33%

%LRT SIG: proportion of datasets with significant likelihood-ratio test, most flexible (%): proportion of datasets where the procedure identified DIF at least on the correct item-covariate pairs (among others), flexible (%): proportion of datasets where the procedure identified DIF on the correct item-covariate pairs only, perfect (%): proportion of datasets were the procedure identified exactly the DIF that was simulated (correct form and correct pairs). Setting No. 1: The two covariates are not correlated and they induce DIF on two distinct items. Setting No. 2: The two covariates are not correlated and they induce DIF on the same item. Setting No. 3: The two covariates are correlated and only one induces DIF on two items. The procedures converged on all datasets. No identifiability issues were encountered. Results are given according to the simulation characteristics: setting, DIF form (homogeneous H, non-homogeneous NH), DIF size, sample size *n*, number of items *J*.

The performance of the three procedures varied depending on the values of the simulation characteristics. First, all procedures failed to detect DIF within scenarios with weak DIF and a sample size of 400: Most Flexible detection rates did not exceed 10% under these conditions. Therefore, results regarding these scenarios will not be further developed. The following paragraphs focus only on the results observed when the sample size *n* equals 800 or when the DIF size is medium (with either *n* = 400 or 800).

#### 3.2.1. Performance within settings Nos. 1 and 2 (uncorrelated covariates both inducing DIF)

Whatever the procedure, Most Flexible detection rates were low when DIF size was weak and *n* = 800 (from 16 to 34%). However, when DIF size was medium, Most Flexible detection rates were moderate when *n* = 400 (between 35 and 56%) and high when *n* = 800 (from 76 to 96%). As a reminder, these rates indicate to what extent the different procedures are able to detect DIF on at least the item-covariate pairs on which DIF was simulated. Best performance regarding Most Flexible detection rates was observed for ROSALI-DIF FORWARD (ranging from 20 to 96%, mean: 56%) but ROSALI-DIF BACKWARD also showed quite similar performance (rates did not differ by more than 5%). The performances of PCMLasso were generally slightly lower (rates ranging from 16 to 90%, mean: 50%). Of note, the three methods showed usually higher Most Flexible detection rates when DIF was non-homogeneous than when it was homogeneous, all other scenario characteristics being equal, with a maximal difference up to +17% (mean difference of +8%).

Both ROSALI-DIF algorithms showed poor Flexible detection rates when DIF size was weak and *n* = 800 (from 13 to 32%), moderate Flexible detection rates when DIF size was medium and *n* = 400 (from 30 to 49%), and high Flexible detection rates when DIF size was medium and *n* = 800 (from 65 to 81% and 78 to 87% for ROSALI-DIF FORWARD and ROSALI-DIF BACKWARD, respectively). Regarding PCMLasso, we observed poor Flexible detection rates in all scenarios except those with a sample size of 800 and medium DIF size where rates ranged from 37 to 54%. Hence, based on the Flexible criteria, the best-performing methods are the ROSALI-DIF algorithms. Of note, ROSALI-DIF BACKWARD outperformed ROSALI-DIF FORWARD when DIF size was medium and *n* = 800 (Flexible detection rates of both methods differed from +5 to +16%) while their performance was similar under other scenarios. Finally, the three methods showed generally higher Flexible detection rates when DIF was non-homogeneous than when it was homogeneous (all other scenario characteristics being equal), with a maximal difference up to +17% (mean difference of +7%).

Flexible detection rates were lower than the Most Flexible detection rates whatever the procedure. It means that, in addition to the correct DIF item-covariate pairs, all procedures wrongly detected other pairs (on which DIF was not simulated). Gaps between the Most Flexible and Flexible detection rates usually increased with increasing Most Flexible detection rates for all procedures (the higher the Most Flexible detection rate, the greater the gap). For both ROSALI-DIF algorithms, gaps also increased with increasing number of items *J*. These gaps were always the smallest for ROSALI-DIF BACKWARD and the largest for PCMLasso.

Among scenarios with homogeneous DIF, ROSALI-DIF algorithms both showed Perfect detection rates close to the Flexible detection rates (e.g., they differed from 4 to 10% for medium DIF scenarios). Thus, within datasets meeting the Flexible criteria, both algorithms correctly determined the form of DIF involved when the simulated DIF was homogeneous. As for PCMLasso, Perfect detection rates did not exceed 2% indicating that PCMLasso failed to identify the correct DIF form when the simulated DIF was homogeneous. Hence, based on the Perfect criterion, both ROSALI-DIF algorithms outperformed PCMLasso within scenarios with homogeneous DIF. However, when the simulated DIF was non-homogeneous, Perfect detection rates associated with ROSALI-DIF algorithms were substantially lower than Flexible detection rates (e.g., gaps ranged from 30 to 56% among scenarios with medium DIF). Hence, both algorithms struggled to identify the correct form of DIF (i.e., non-homogeneous) among scenarios meeting the Flexible criteria. On the contrary, PCMLasso showed Perfect detection rates very close to the Flexible detection rates (i.e., rates ranging from 49 to 53% when DIF was medium and *n* = 800, low rates ranging from 12 to 26% otherwise). It indicates that once PCMLasso correctly identified the item-covariate pairs affected by DIF, it also correctly identified the DIF form. Therefore, when DIF was non-homogeneous, the PCMLasso approach showed larger Perfect detection rates than the ROSALI-DIF algorithms. Based on the Perfect criterion, PCMLasso outperformed ROSALI-DIF algorithms when the simulated DIF was non-homogeneous. Note that, as observed for Flexible detection rates, ROSALI-DIF BACKWARD showed higher Perfect detection rates than ROSALI-DIF FORWARD in scenarios with medium DIF and *n* = 800. Both algorithms showed similar performance otherwise.

#### 3.2.2. Performance within setting No. 3 (correlated covariates with only one inducing DIF)

Among scenarios of setting No. 3, the performance of the three DIF detection methods were usually poorer as compared to settings Nos. 1 and 2 for all criteria.

Indeed, regarding the Most flexible and Flexible detection rates, the performance of all three methods was globally poor when DIF size was weak or when the sample size equaled 400. For both ROSALI-DIF algorithms, these rates increased among scenarios with n = 800 and medium DIF (ranging from 69 to 86% for the Most Flexible detection rates and from 61 to 80% for the Flexible detection rate). Performance was poorer for PCMLasso under the same conditions as Most Flexible and Flexible detection rates ranged from 19 to 78% and from 3 to 34%, respectively. Of note, we observed similar effects to those highlighted in settings Nos. 1 and 2 regarding these rates and the associated gaps.

### 3.3. Bias and empirical standard error – DIF scenarios

Bias, empirical standard errors, and average model standard errors associated with the estimation of β_1_ and β_2_ (the respective effect of the covariates *C*_*1*_ and *C*_*2*_ on the latent variable level) are given in Supplementary Appendix D. Under settings Nos. 1 and 2, bias remained small for all methods; it never exceeded 0.08 in absolute value. Under setting No. 3 results were more mixed: bias related to the estimation of β_1_ (the effect of *C*_*1*_, the only DIF-inducing covariate in this setting) remained small when *J* = 7 but it increased when *J* = 4 for all procedures (reaching −0.19 for ROSALI-DIF algorithms and −0.14 for PCMLasso). Across all settings, the better the methods detected DIF, the lower the bias.

### 3.4. DIF parameter estimates

On the one hand, PCMLasso always underestimated the DIF parameters $\gamma_{j\,p}^{\left(C\right)}$. On the other hand, DIF parameters estimates were much closer to the true simulated values for ROSALI-DIF algorithms but they showed a larger dispersion (see the boxplots available through the R Shiny app, Supplementary Appendix E).

## 4. Discussion

### 4.1. Main results

This study aimed to extend the first part of the ROSALI algorithm (dedicated to DIF detection at one time point using RMT) in order to consider two binary covariates instead of one. We proposed two extensions: ROSALI-DIF FORWARD and ROSALI-DIF BACKWARD. The novelty characterizing these extensions is the screening step that aims to identify the item-covariate pairs candidate for DIF detection and the item-covariate pairs that will be considered as anchors (i.e., not affected by DIF). This further step was inspired by the iterative Wald test procedure proposed by Tay et al. (2015) and Cao et al. (2017). These authors indicated that testing all items for DIF in a fully unconstrained model had good power but a high Type I error, so it could be useful for identifying anchor items in a preliminary stage (Tay et al., 2015; Cao et al., 2017). Performance of each extension of ROSALI for DIF detection were assessed by simulations alongside the performance of the approach based on likelihood penalization proposed by Schauberger and Mair (2020) (i.e., PCMLasso) under conditions that aimed to be representative of health research contexts.

In light of the rates of false detection of DIF, both ROSALI-DIF algorithms satisfactorily prevent from inferring DIF when it has not been simulated. This good performance may be explained by: (i) combining the LRT performed at a 5% significance level with the screening and iterative steps, and (ii) the Bonferroni correction applied during the iterative step. In light of the Flexible and Most Flexible detection rates, both ROSALI-DIF algorithms can detect item-covariate pairs having medium DIF (as simulated in this manuscript) with good power for studies with two correlated or uncorrelated binary covariates, a sample size of 800, and a questionnaire similar to the ones simulated with regards to *M* and *J*. Moreover, ROSALI-DIF algorithms should generally not wrongly detect items-covariates pairs without DIF. However, one must be cautious regarding the form of DIF evidenced by these algorithms, as non-homogenous DIF is rarely identified as such. It means that the test performed during step 5 generally lacked power as it failed to reject the null hypothesis of homogeneous DIF when DIF was actually non-homogenous. Correct identification of the DIF form may require larger sample sizes.

Regarding PCMLasso, rates of false detection of DIF among scenarios without DIF were high. Indeed, PCMLasso was prone to erroneously detect DIF in at least one item-covariate pair in almost half of the datasets, no matter the scenario. This drawback is also highlighted by the large gaps between the Flexible and Most flexible detection rates among scenarios where DIF was simulated. Indeed, PCMLasso was likely to wrongly flag other item-covariate pairs in addition to the ones truly affected by DIF. However, we noticed that the estimated size of the wrongly evidenced DIF effects remained small on average, which did not result in a meaningful measurement bias at the scale level (data not shown in the manuscript but available on OSF, see the data availability statement). One must be cautious regarding the form of DIF evidenced by this approach. Indeed, it almost always suspected the occurrence of non-homogeneous DIF (whatever the form of DIF simulated). After further investigations, we also noticed that in some datasets, DIF parameters estimates were very close (e.g., $\hat{\gamma_{j\,1}^{\left(C\right)}}$ = 0.28 and $\hat{\gamma_{j\,2}^{\left(C\right)}}$ = $\hat{\gamma_{j\,3}^{\left(C\right)}}$ = 0.29), indicating that the tuning parameter may have been chosen just after the split in the DIF parameters path (see the border between the “Homogeneous DIF” area and the “Non-homogeneous DIF” area pictured in Figure 4A).

All three methods showed small capacity to detect weak DIF. It may not be a major issue as the weak DIF simulated in this simulation study might generally not result in a meaningful measurement bias at the scale level. Moreover, DIF detection performance of ROSALI-DIF algorithms and PCMLasso decreased with decreasing sample size. This effect was expected but is sharply marked and may be even more problematic with unbalanced covariates.

The evaluation criteria we used to evaluate the DIF detection performance of the three procedures are not so common in the literature on DIF detection. More frequent evaluation criteria are the average false-positive and true-positive rate (FPR and TPR, respectively). However, the focus of these criteria is different from the criteria we used:

- Regarding DIF effects erroneously identified:
  On the one hand, the rate of false DIF detection that we used in our simulation study quantifies the risk of drawing an erroneous conclusion when performing the procedure on a given dataset where no DIF was simulated (i.e., inferring the presence of DIF while DIF was not simulated in the dataset). It does not quantify whether there are many item-covariate pairs flagged for DIF among the datasets in which DIF was wrongly inferred. On the other hand, the average FPR quantifies whether or not many item-covariate pairs are incorrectly flagged for DIF among the datasets without DIF. However, it does not inform about the proportion of datasets where the adequate conclusion was arrived at (that is: no DIF is present within the set of items considered).
- Regarding DIF effects correctly identified:
  The rate of correct DIF detection (with the Most Flexible, Flexible or Perfect criteria) quantifies the probability of reaching the right conclusion when performing the procedure on a given dataset where DIF was simulated (i.e., inferring the presence of DIF and correctly identifying the DIF item-covariate pairs). As for the average TPR, it indicates whether the DIF item-covariate pairs are often rightly flagged for DIF, but it does not inform about the proportion of datasets where the adequate conclusion was arrived at (that is: DIF is present within the set of items considered and the correct item-covariate pairs are flagged).

The estimation of the average FPR and TPR was not planned in the initial aims of our simulation study, but since they may interest readers, they are provided in Supplementary Appendix F.

The DIF parameters $\gamma_{j\,p}^{\left(C\right)}$, were satisfactorily recovered for ROSALI-DIF algorithms among datasets meeting the Most Flexible criteria. Conversely, the PCMLasso approach always underestimated them due to the penalization that shrinks them toward zero. This downward bias has already been previously highlighted (Tutz and Schauberger, 2015; Schauberger and Mair, 2020). Of note, Tutz and Shauberger indicated that the bias introduced by the parameter shrinkage could be removed by an additional refit (i.e., fitting a final unpenalized model that only includes DIF effects evidenced after the likelihood penalization approach).

Finally, all procedures provided a globally unbiased estimation of the effect of the covariates on the latent variable level adjusted for DIF. Besides, the better the methods detected DIF, the lower the bias. Among scenarios with DIF simulated under settings Nos. 1 and 2, bias remained small for all methods (even when DIF was weak, a condition under which the procedures all showed low performance). This observation of globally unbiased estimations of β_1_ and β_2_ when DIF was weak (despite the procedures’ low performance) could mean that such a DIF condition does not trigger meaningful bias if DIF is not accounted for. Among scenarios with DIF simulated under setting No. 3, results were more mixed: bias related to the estimation of β_1_ (the effect of *C*_*1*_, the only DIF-inducing covariate in this setting) remained small when *J* = 7 but it increased when *J* = 4. This latter condition corresponds to a test composed of four items and half of them affected by DIF induced by a single covariate. As demonstrated by Rouquette et al. (2016) in a simulation study, such a configuration may lead to a meaningful bias if DIF is ignored, even if DIF is weak. Hence, the fact that the three procedures showed generally lower DIF detection performance among these scenarios may be one of the causes of these biased estimates. Of note, DeMars and Lau reported that DIF is conceptualized as differences in the item endorsement probabilities after controlling the psychological variable or capacity targeted by the questionnaire (DeMars and Lau, 2011). Yet, if a large proportion of items is affected by DIF (i.e., ≥ 50%), then the questionnaire might measure different constructs among the groups being compared, and it would make no sense to speak about controlling the psychological variable level or ability (DeMars and Lau, 2011) as the target construct may not be conceptualized in the same way across groups.

To summarize, the DIF detection methods presented in the manuscript allow to simultaneously model the DIF effects from different covariates. They are useful when one wants to grasp the potential multiple sources of DIF, especially when these sources are correlated. Indeed, in this latter case, such DIF detection methods should be preferred to the strategy that consists in performing the DIF detection independently for each covariable (one-covariate-at-a-time analysis). However, we can see that the three methods considered require a large enough sample size, as their DIF detection performance was low to moderate when DIF size was medium and *n* = 400. Between the three methods, ROSALI-DIF algorithms (FORWARD and BACKWARD) seemed to be preferable if one aims to correctly identify the item-covariate pairs affected by DIF. A Stata module automating both algorithms is in preparation. Of note, all the methods evaluated within this manuscript were based on Rasch measurement theory. Hence, they assume that the assumptions of RMT modeling are met, that is: unidimensionality, local independence, monotonicity, and items all equally indicative of the latent variable. In practice, the adequacy of Rasch modeling should be investigated prior to conducting DIF detection.

### 4.2. Limitations and perspectives

Several limitations can be addressed. First, within our simulation study, the differences observed between DIF scenarios with *J* = 4 or 7 items could either be due to: (i) an increase in the number of items, (ii) a decrease in the proportion of true DIF items, or (iii) a decrease in the proportion of item-covariate pairs on which DIF was simulated. Further investigations are hence needed to disentangle the effects of these different simulation parameters.

In addition, neither ROSALI extensions nor the PCMLasso approach allows to consider that the DIF effect of one covariate may depend on the level of another covariate. To allow for such a phenomenon, it would be necessary to introduce an interaction term between the covariates. Yet, more developments are needed to know how to deal with such an interaction. Of note, the PCM-IFT approach systematically models such an interaction because of its philosophy of recursive partitioning. More broadly, it would be worth comparing our results with the performance of the PCM-IFT approach under the same conditions when it will be able to account for the effect of a covariate on the latent variable.

Besides, we have considered simple situations with two well-balanced covariates that did not impact the latent variable level. Extending the simulation framework with: (i) less balanced covariates, (ii) covariates with an impact on the latent variable level, and (iii) more than two covariates, would be interesting to have more insight into the performance of the evaluated procedures. In addition, it would be worth evaluating these procedures under a wider DIF setting (e.g., considering different DIF directions within one scenario). Finally, all datasets were complete: there were no missing data with regards to item responses nor covariate. A simulation study with incomplete datasets could be of value to evaluate the performance of the DIF detection methods, in a situation more representative of real data. Of note, as parameters are estimated using marginal maximum likelihood, estimations should be asymptotically unbiased in case of incomplete data missing (completely) at random. Nonetheless, due to a loss of precision of the estimations, a loss in the performance can be expected.

The methods evaluated in this manuscript are based on statistical results. Hence, it is critical to ensure that the evidenced DIF effects are relevant and meaningful. Regarding the ROSALI-DIF algorithms, they are more specifically based on statistical testing. One drawback of such an approach is that they are likely to detect minor DIF effects as soon as the sample is large enough. An alternative could be to use DIF effect size to evaluate whether a given difference in item parameters is substantial or negligible (Henninger et al., 2023). Besides, it could be interesting to add *a priori* clinical knowledge on the items on which DIF may be likely (depending on the patients’ population) in order to not only rely on statistical criteria.

All these concerns about: (i) choosing which covariates to investigate for DIF, (ii) adding prior knowledge to the analysis, (iii) using univariate pre-selection (i.e., one-covariate-at-a-time analysis) before multivariable modeling, and (iv) using forward and backward strategies are broader concerns that do not pertain to DIF detection; they are encountered in all regression modeling strategies. Of note, forward and backward strategies are traditional and straightforward way to filter out predictors of a given outcome from a pool of candidates containing both true predictors and noise variables. Although widespread, these approaches show disadvantages that have been extensively addressed in the regression modeling literature (Steyerberg, 2009; Harrell, 2015). Among others, we can mention statistical inference issues and the fact that these approaches sometimes fail to filter candidate variables correctly: they are known to select noise variables and drop true predictors, especially when the set of candidate predictors is large and/or in the presence of collinearity (Derksen and Keselman, 1992; Steyerberg, 2009). Although likelihood penalization is often presented as a promising alternative, we found that PCMLasso’s performance was not better than the ROSALI algorithms’ performance in our simulation framework.

Further developments are needed to better grasp the sources and determinants of lack of measurement invariance in health research. To that end, DIF detection methods allowing considering several covariates simultaneously (with two categories or more) could be of great practical interest.

## Data availability statement

The datasets presented in this study can be found in online repositories. The names of the repository/repositories and accession number(s), simulated datasets, modules, and scripts related to this simulation study are openly available at: https://osf.io/hkp38/?view_only=81c3c3c42e194760a7accbd7cc9b431f.

## Author contributions

YD simulated and analyzed the data and drafted the manuscript. MB, VS, and J-BH revised substantially the manuscript. All authors participated in the development of this study, contributed to the manuscript revision, read, and approved the submitted version.

## Footnotes

1 Of note, as we used RMT to simulate and analyze the data, items were only characterized by item threshold parameters (as there are no other item parameters in Rasch modeling). DIF could therefore only affect these parameters.
