# Supplementary material for: Identification of sources of DIF using covariates in patient-reported outcome measures: a simulation study comparing two approaches based on Rasch family models
Source: Front Psychol. 2023 Aug 10;14:1191107. doi: 10.3389/fpsyg.2023.1191107 (PMC10448192; doi:10.3389/fpsyg.2023.1191107)
Supplement: Supplementary file 1 [file Table_1.DOCX]

# Title: Identification of sources of DIF using covariates in patient-reported outcome measures: a simulation study comparing two approaches based on Rasch family models

**Caption:**

This online resource provides further information regarding the manuscript entitled " Identification of sources of DIF among patient-reported outcome measures using covariates: a simulation study comparing two approaches based on Rasch family models". It is structured as follow:

**Appendix A:** Description of the ROSALI-DIF BACKWARD algorithm

*Step by step presentation of the algorithm and associated statistical considerations*

**Appendix B:** Additional information regarding the simulation study

*- Item threshold parameters used in the simulation study
- Simulation of the correlation between the two covariates*

**Appendix C:** DIF detection performance of ROSALI-DIF BACKWARD among the simulated datasets

*Rates of false detection of DIF and rates of correct DIF detection*

**Appendix D:** Estimation of the covariates’ effect on the latent variable level for the three DIF detection methods

*Bias, empirical standard error and average model standard error associated with the estimation of* $\beta_{k}$*(the effect of the covariate* $C_{k ; k=1,2}$ *on the latent variable level) for both ROSALI-DIF algorithms and PCMLasso*

**Appendix E:** Shiny app associated with the simulation study

*An interactive app that allows to:*

- *Investigate the DIF parameters estimates for ROSALI-DIF algorithms and PCMLasso*
- *Investigate bias, empirical standard error and average model standard error associated with the estimation of* $\beta_{k}$*(the effect of the covariate* $C_{k ; k=1,2}$ *on the latent variable level) for both ROSALI-DIF algorithms and PCMLasso*

**Appendix F:** Average false-positive and true-positive rates

**Appendix A:** Description of the ROSALI-DIF BACKWARD algorithm

**Table A1:** Comprehensive description of the ROSALI-DIF BACKWARD algorithm and statistical considerations

| **ROSALI-DIF BACKWARD steps** | **Statistical considerations** |
| --- | --- |
| **Step 1: Estimation of a fully non-invariant model (Model A)**  *Same as ROSALI-DIF FORWARD* | *Same as ROSALI-DIF FORWARD* |
| **Step 2: Estimation of a fully invariant model (Model B)**  *Same as ROSALI-DIF FORWARD* | *Same as ROSALI-DIF FORWARD* |
| **Step 3. Test of the global occurrence of DIF through a likelyhood-ratio test**  *Same as ROSALI-DIF FORWARD* | *Same as ROSALI-DIF FORWARD* |
| **Step 4. Screen item-covariate pairs (Item**$\boldsymbol{j}\mathbf{,}$ **Covariate** $\boldsymbol{C}\mathbf{)}$ **candidate for DIF detection**  *Same as ROSALI-DIF FORWARD* | *Same as ROSALI-DIF FORWARD* |
| **Step 5. Selection of DIF item-covariate pairs (Item**$\boldsymbol{j}\mathbf{,}$ **Covariate** $\boldsymbol{C}\mathbf{)}$ **among candidates**  This step is an iterative step that aims at selecting pairs affected by DIF among candidate pairs. A new model (called Model C) is introduced. This model is based on Model B, but invariance constraints are relaxed for all candidate pairs (i.e., those associated with a significant test during step 4). From model C, tests for joint hypothesis are performed for each candidate pair to determine whether the DIF effect induced by covariate $C$ on item $j$ is significant or not. If all candidate pairs are associated with a significant test after a Bonferroni correction, then occurrence of DIF will be assumed on all these pairs and the algorithm moves to the next step. Otherwise, model C is updated so that the pairs associated with non-significant tests are switched to anchors (they will no longer be considered as candidate for DIF detection)  Step 5 is repeated until all remaining candidate pairs are significant or until there is no candidate pair left (no DIF detected). Of note, if no anchor items are identified for one of the covariates (or the two of them), the algorithm ends with an error message of non-identifiability. | ********* Test DIF effect of candidate pairs *********  **Null and alternative hypotheses of contrast test for DIF:**  $H_{0})$ $\forall p, \gamma_{jp}^{(C)}$ = 0 (No DIF)  $H_{1}$) $\exists p : \gamma_{jp}^{(C)}\neq$ 0 (DIF)  **Significance level**:  5%/number of candidate pairs, Bonferroni correction performed to avoid the inflation of the type I error rate due to multiple testing.  ************** Update Model C *****************  Invariance constraints are added for the pairs associated with non-significant tests so that they are considered as anchor pairs |
| **Step 5bis. Assessment of the form of DIF involved**  For each DIF-pair selected at step 5, the involved form of DIF is assessed using a test performed on model C estimates.  Once all DIF-pairs have been tested, model C is updated to account for the evidenced DIF form. | ******* Test DIF form on the selected pairs *********  **Null and alternative hypotheses of contrast test to assess DIF form:**  $H_{0}$ : $\forall p, \gamma_{j^{*}p}^{(C^{*})}$ = $\gamma_{j^{*}}^{(C^{*})}$ (homogeneous)   $H_{1}$ : $\exists p, p' : \gamma_{j^{*}p}^{(C^{*})}\neq\gamma_{j^{*}p^{'}}^{(C^{*})}$ (non-homogeneous)  **Significance level:** 5%  ************** Update Model C *****************  For all DIF-pairs associated with a significant test, the associated DIF parameters $\gamma_{j^{*}p}^{(C^{*})}$ are freely estimated (non-homogeneous DIF). Otherwise, for DIF-pairs associated with a non-significant test, the DIF parameters $\gamma_{j^{*}p}^{(C^{*})}$ are estimated but constrained to be constant over the response categories (homogeneous DIF). |
| **Step 6. Estimation of the covariates effect on the latent variable level (Model D)**  The last step estimates the effect of the covariates $C_{1}$ and $C_{2}$ on the latent variable level adjusted for the DIF that was previously evidenced, if appropriate, using a final model called model D. | Model D = Model B if no DIF has been evidenced. Model D is based on the last version of model C (step 5bis), where forms of DIF have been determined. |
| *Note: This algorithm estimates several PCMs derived from Equation 2 with marginal maximum likelihood estimation. For all PCMs, common variance of the latent variable distribution will be assumed.* | |

**Appendix B:** Additional information regarding the simulation study

*Item threshold parameters used in the simulation study*

For each item $j$, the threshold parameter of the first positive response category (denoted $\delta_{j1}$) equaled the $\frac{j}{J+1}th$quantile from a $\mathcal{N}\left( 0,1 \right)$. Threshold parameters of the following response categories were then regularly shifted from the first one: $\delta_{jp}=\delta_{j1}+\left( p-1 \right)\times\frac{2}{M-2}$ , $p=2,\ldots,M-1$. Finally, threshold parameters of all items were centered by subtracting the mean $\bar{\delta}=\frac{\sum_{j,p} \delta_{jp}}{J(M-1)}$. Thus, all item threshold parameters were centered on the same mean as the latent variable distribution. Item thresholds used for the simulations are summarized in Table B1.

**Table B1:** Item threshold parameters used for the simulation study

|  | **Item thresholds** | | |
| --- | --- | --- | --- |
|  | $\delta_{j1}$ | $\delta_{j2}$ | $\delta_{j3}$ |
| **Scenarios with** $\boldsymbol{J=4}$ |  |  |  |
| Item 1 | -1.84 | -0.84 | 0.16 |
| Item 2 | -1.25 | -0.25 | 0.75 |
| Item 3 | -0.75 | 0.25 | 1.25 |
| Item 4 | -0.16 | 0.84 | 1.84 |
| **Scenarios with** $\boldsymbol{J=7}$ |  |  |  |
| Item 1 | -2.15 | -1.15 | -0.15 |
| Item 2 | -1.67 | -0.67 | 0.33 |
| Item 3 | -1.32 | -0.32 | 0.68 |
| Item 4 | -1.00 | 0.00 | 1.00 |
| Item 5 | -0.68 | 0.32 | 1.32 |
| Item 6 | -0.33 | 0.67 | 1.67 |
| Item 7 | 0.15 | 1.15 | 2.15 |
| $J$: Number of items  $\delta_{jp}$ difficulty of response category $p$ of item $j$ | | | |

*Formulas for DIF-items threshold parameters*

In the setting No. 1, if the covariate $C_{k}$ ($k=1$ or $2)$ induced DIF on item $j$, then associated threshold parameters were given by:

$$\delta_{jp}+\gamma_{jp}^{(C_{k})}\times C_{k}= \left\{ \begin{matrix} \delta_{jp} \text{in the group} C_{k} = 0 \\ \delta_{jp}+\gamma_{jp}^{(C_{k})} \text{in the group} C_{k} = 1 \end{matrix} \right.$$

In the setting No. 2, the formula is different, as DIF occurred on the same item for the two covariates. The threshold parameters of the DIF-item were given by:

$$\delta_{jp}+\gamma_{jp}^{(C_{1})}\times C_{1}+\gamma_{jp}^{(C_{2})}\times C_{2}= \left\{ \begin{matrix} \delta_{jp} \text{in the group} C_{1}= {0 \wedge C}_{2} = 0 \\ \delta_{jp}+\gamma_{jp}^{(C_{1})} \text{in the group} C_{1}= {1 \wedge C}_{2} = 0 \\ \delta_{jp}+\gamma_{jp}^{(C_{2})} \text{in the group} C_{1}={0 \wedge C}_{2} = 1 \\ \delta_{jp}+\gamma_{jp}^{(C_{1})}+\gamma_{jp}^{(C_{2})} \text{ in the group} C_{1}= {1 \wedge C}_{2} = 1 \end{matrix} \right.$$

In the setting No. 3, if the covariate $C_{1}$ induced DIF on item $j$, then associated threshold parameters were given by:

$$\delta_{jp}+\gamma_{jp}^{(C_{1})}\times C_{1}= \left\{ \begin{matrix} \delta_{jp} \text{in the group} C_{1} = 0 \\ \delta_{jp}+\gamma_{jp}^{(C_{1})} \text{in the group} C_{1} = 1 \end{matrix} \right.$$

*Correlations between covariates*

For settings where the two covariates $C_{1}$ and $C_{2}$ were not correlated, the counts in the cross-tabulation were well balanced and equaled the sample size ($n$) divided by 4:

|  |  | **Covariate** $\boldsymbol{C}_{\boldsymbol{1}}$ | |  |
| --- | --- | --- | --- | --- |
|  |  | 0 | 1 | **Total** |
| **Covariate** $\boldsymbol{C}_{\boldsymbol{2}}$ | 0 | $n/4$ | $n/4$ | $n/2$ |
|  | 1 | $n/4$ | $n/4$ | $n/2$ |
| **Total** | | $n/2$ | $n/2$ | $n$ |


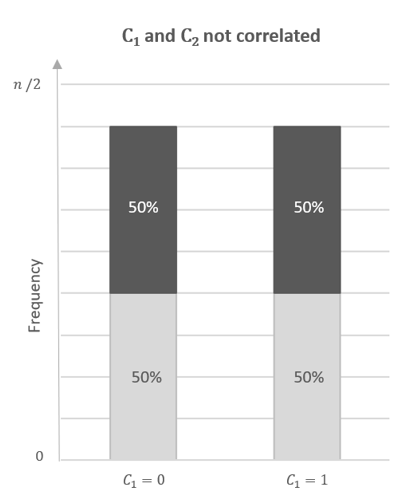

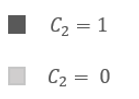

$$n/2$$

We unbalanced these counts to simulate the correlation between the two covariates. Specifically, we increased the counts in the diagonal:

|  |  | **Covariate** $\boldsymbol{C}_{\boldsymbol{1}}$ | |  |
| --- | --- | --- | --- | --- |
|  |  | 0 | 1 | **Total** |
| **Covariate** $\boldsymbol{C}_{\boldsymbol{2}}$ | 0 | $3n/8$ | $n/8$ | $n/2$ |
|  | 1 | $n/8$ | $3n/8$ | $n/2$ |
| **Total** | | $n/2$ | $n/2$ | $n$ |


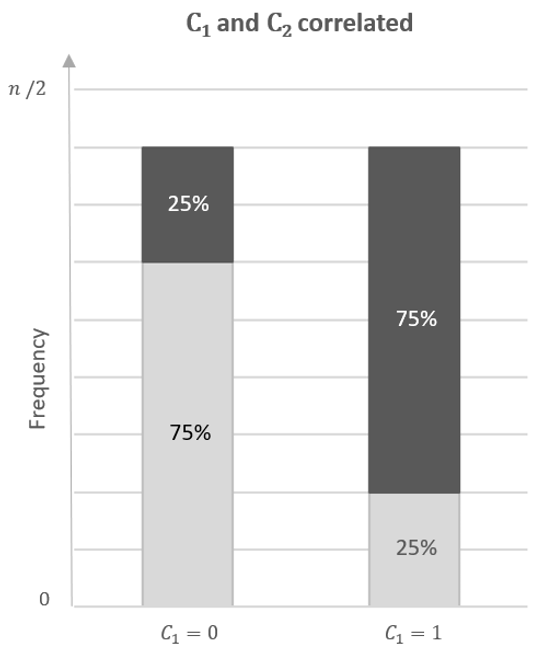

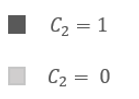

$$n/2$$

**Appendix C:** DIF detection performance of ROSALI-DIF BACKWARD among the simulated datasets

*Rates of false DIF detection – No DIF scenarios*

**Table C1:** Rates of false detection of DIF (%DIF Detected) and rates of significant likelihood-ratio tests (%LRT SIG) among scenarios with no simulated DIF for the ROSALI-DIF BACKWARD algorithm. Rates of false detection of DIF are computed at the end of procedure.
Results are given according to the simulation characteristics $n$(sample size), $J$ (number of items) and the presence or absence of correlation between covariates $C_{1}$ and $C_{2}$.

|  |  |  | **ROSALI-DIF BACKWARD** | |
| --- | --- | --- | --- | --- |
| $\boldsymbol{n}$ | ***J*** | **Corr** | **%LRT**  **SIG** | **%DIF  wrongly detected** |
| 400 | 4 | No | 5% | 4% |
| 400 | 4 | Yes | 6% | 3% |
| 400 | 7 | No | 7% | 6% |
| 400 | 7 | Yes | 6% | 4% |
| 800 | 4 | No | 6% | 4% |
| 800 | 4 | Yes | 5% | 4% |
| 800 | 7 | No | 6% | 5% |
| 800 | 7 | Yes | 4% | 3% |
| **%DIF wrongly detected:** Proportion of datasets where DIF was wrongly detected on at least one item-covariate pair at the end of the procedure (i.e., rate of false DIF detection)  **%LRT SIG:** Proportion of datasets with a significant likelihood-ratio test  **Corr:** Correlation, indicates whether covariates $C_{1}$ and $C_{2}$ are correlated (=Yes) or not (=No)  *Note: ROSALI-DIF BACKWARD converged on all datasets. No identifiability issues were encountered* | | | | |

*Rates of correct DIF detection – DIF scenarios*

**Table C2:** Rates of correct detection of DIF among scenarios with simulated DIF for the ROSALI-DIF BACKWARD algorithm. Rates are computed at the end of the procedure. Results are given according to the following simulation characteristics: DIF form, DIF size, sample size $n$ and number of items *J*.

|  |  |  |  | **ROSALI-DIF BACKWARD** | | | | | | | | | | | |
| --- | --- | --- | --- | --- | --- | --- | --- | --- | --- | --- | --- | --- | --- | --- | --- |
|  |  |  |  | **Setting No. 1** | | | | **Setting No. 2** | | | |  | **Setting No. 3** | | |
| **DIF form** | **DIF size** | $\boldsymbol{n}$ | ***J*** | **%LRT**  **SIG** | **Most  flexible** | **Flexible** | **Perfect** | **%LRT**  **SIG** | **Most  flexible** | **Flexible** | **Perfect** | **%LRT**  **SIG** | **Most  flexible** | **Flexible** | **Perfect** |
| Homogeneous | Weak | 400 | 4 | 31% | 3% | 3% | 1% | 29% | 2% | 1% | 1% | 21% | 3% | 2% | 1% |
| Homogeneous | Weak | 400 | 7 | 27% | 5% | 4% | 3% | 26% | 2% | 2% | 1% | 23% | 2% | 1% | 1% |
| Homogeneous | Weak | 800 | 4 | 64% | 16% | 15% | 12% | 68% | 20% | 19% | 16% | 44% | 8% | 7% | 6% |
| Homogeneous | Weak | 800 | 7 | 63% | 24% | 21% | 17% | 58% | 19% | 16% | 12% | 44% | 13% | 11% | 9% |
| Homogeneous | Medium | 400 | 4 | 85% | 36% | 33% | 29% | 83% | 38% | 36% | 31% | 58% | 20% | 20% | 16% |
| Homogeneous | Medium | 400 | 7 | 79% | 39% | 35% | 30% | 82% | 39% | 33% | 28% | 67% | 26% | 24% | 19% |
| Homogeneous | Medium | 800 | 4 | 99% | 88% | 81% | 73% | 99% | 89% | 84% | 74% | 91% | 71% | 66% | 58% |
| Homogeneous | Medium | 800 | 7 | 99% | 91% | 81% | 73% | 99% | 92% | 78% | 73% | 96% | 77% | 70% | 62% |
|  |  |  |  |  |  |  |  |  |  |  |  |  |  |  |  |
| Non-homogeneous | Weak | 400 | 4 | 33% | 4% | 4% | 0% | 34% | 4% | 4% | 1% | 24% | 3% | 2% | 0% |
| Non-homogeneous | Weak | 400 | 7 | 31% | 4% | 3% | 0% | 32% | 6% | 6% | 1% | 28% | 2% | 1% | 0% |
| Non-homogeneous | Weak | 800 | 4 | 68% | 25% | 23% | 3% | 74% | 30% | 29% | 2% | 48% | 13% | 13% | 1% |
| Non-homogeneous | Weak | 800 | 7 | 62% | 24% | 21% | 3% | 67% | 29% | 25% | 3% | 52% | 17% | 15% | 2% |
| Non-homogeneous | Medium | 400 | 4 | 91% | 51% | 49% | 6% | 86% | 46% | 43% | 6% | 73% | 33% | 30% | 5% |
| Non-homogeneous | Medium | 400 | 7 | 79% | 48% | 41% | 6% | 79% | 48% | 42% | 7% | 75% | 36% | 32% | 6% |
| Non-homogeneous | Medium | 800 | 4 | 100% | 94% | 87% | 35% | 100% | 93% | 86% | 30% | 98% | 85% | 80% | 31% |
| Non-homogeneous | Medium | 800 | 7 | 100% | 95% | 80% | 30% | 99% | 95% | 81% | 28% | 98% | 85% | 74% | 29% |
| **%LRT SIG:** Proportion of datasets with significant likelihood-ratio test **Most flex (%):** Proportion of datasets where the procedure identified DIF at least on the correct item-covariate pairs (among others)  **Flex (%):** Proportion of datasets where the procedure identified DIF on the correct item-covariate pairs only  **Perfect (%):** Proportion of datasets where the procedure identified exactly the DIF that was simulated (correct form and correct pairs)  **Setting No. 1:** The two covariates are not correlated and they induce DIF on two distinct items  **Setting No. 2:** The two covariates are not correlated and they induce DIF on the same item  **Setting No. 3:** The two covariates are correlated and only one induces DIF on two items  *Note: ROSALI-DIF BACKWARD converged on all datasets. No identifiability issues were encountered.* | | | | | | | | | | | | | | | |

**Appendix D:** Estimation of the covariates’ effect on the latent variable level for the three DIF detection methods

We determined the bias in the estimation of the covariates' effects on the latent variable level (denoted $\beta_{1}$ and $\beta_{2}$ for the covariates $C_{1}$ and $C_{2}$, respectively) to determine whether the three methods enable for an unbiased estimation after DIF detection. Bias in $\beta_{k (k=1, 2)}$ estimation was computed for each scenario as the mean error, that is $E\left[ \hat{\beta_{k}} \right]-\beta_{k}$, where$\beta_{k}$is the true simulated value for the effect of covariate $C_{k}$ on the latent variable level and $\hat{\beta_{k}}$ stands for the estimates of $\beta_{k}$ among the replications of the considered scenario. Of note, for all scenarios $\beta_{1}$ and $\beta_{2}$ were set to 0.

In addition to bias, we computed the standard deviation of the $\beta_{k (k=1, 2)}$ estimates, i.e. $\sqrt{Var(\hat{\beta_{k}})}$, which is also known as empirical standard error.

Empirical standard errors can be compared to the average model standard errors (computed as $\sqrt{E\left[ \hat{Var}\left( \hat{\beta_{k}} \right) \right]}$) which are only available for both ROSALI-DIF algorithms.

|  |  |  |  |  | **ROSALI-DIF** | | **ROSALI-DIF  BACKWARD** | | **PCMLasso** | |
| --- | --- | --- | --- | --- | --- | --- | --- | --- | --- | --- |
|  |  |  |  |  | $\boldsymbol{\beta}_{\boldsymbol{1}}$ | $\boldsymbol{\beta}_{\boldsymbol{2}}$ | $\boldsymbol{\beta}_{\boldsymbol{1}}$ | $\boldsymbol{\beta}_{\boldsymbol{2}}$ | $\boldsymbol{\beta}_{\boldsymbol{1}}$ | $\boldsymbol{\beta}_{\boldsymbol{2}}$ |
| **Setting** | **DIF  form** | **DIF  size** | $\boldsymbol{n}$ | **J** | **Bias (EmpSE)** | **Bias (EmpSE)** | **Bias (EmpSE)** | **Bias (EmpSE)** | **Bias (EmpSE)** | **Bias (EmpSE)** |
| 1 | H | Weak | 400 | 4 | -0.07 (0.12) | -0.07 (0.12) | -0.07 (0.12) | -0.07 (0.12) | -0.04 (0.06) | -0.04 (0.06) |
| 1 | H | Weak | 400 | 7 | -0.04 (0.11) | -0.04 (0.11) | -0.04 (0.11) | -0.04 (0.11) | -0.02 (0.06) | -0.02 (0.06) |
| 1 | H | Weak | 800 | 4 | -0.05 (0.09) | -0.05 (0.09) | -0.05 (0.09) | -0.05 (0.09) | -0.04 (0.05) | -0.03 (0.05) |
| 1 | H | Weak | 800 | 7 | -0.02 (0.08) | -0.02 (0.08) | -0.02 (0.08) | -0.02 (0.08) | -0.02 (0.04) | -0.02 (0.04) |
| 1 | H | Medium | 400 | 4 | -0.05 (0.13) | -0.04 (0.13) | -0.05 (0.13) | -0.05 (0.13) | -0.05 (0.06) | -0.05 (0.06) |
| 1 | H | Medium | 400 | 7 | -0.03 (0.11) | -0.03 (0.12) | -0.03 (0.11) | -0.03 (0.12) | -0.03 (0.05) | -0.03 (0.06) |
| 1 | H | Medium | 800 | 4 | -0.02 (0.09) | -0.01 (0.09) | -0.02 (0.09) | -0.01 (0.09) | -0.04 (0.05) | -0.03 (0.05) |
| 1 | H | Medium | 800 | 7 | -0.00 (0.08) | -0.00 (0.07) | -0.00 (0.08) | -0.00 (0.07) | -0.02 (0.04) | -0.02 (0.04) |
|  |  |  |  |  |  |  |  |  |  |  |
| 1 | NH | Weak | 400 | 4 | -0.07 (0.13) | -0.07 (0.13) | -0.07 (0.13) | -0.07 (0.13) | -0.04 (0.06) | -0.04 (0.06) |
| 1 | NH | Weak | 400 | 7 | -0.03 (0.11) | -0.04 (0.12) | -0.03 (0.11) | -0.04 (0.12) | -0.02 (0.06) | -0.02 (0.06) |
| 1 | NH | Weak | 800 | 4 | -0.04 (0.09) | -0.04 (0.08) | -0.04 (0.09) | -0.04 (0.09) | -0.03 (0.04) | -0.03 (0.04) |
| 1 | NH | Weak | 800 | 7 | -0.01 (0.08) | -0.03 (0.08) | -0.01 (0.08) | -0.03 (0.08) | -0.01 (0.04) | -0.02 (0.04) |
| 1 | NH | Medium | 400 | 4 | -0.02 (0.14) | -0.02 (0.13) | -0.02 (0.14) | -0.02 (0.13) | -0.04 (0.07) | -0.04 (0.06) |
| 1 | NH | Medium | 400 | 7 | -0.01 (0.11) | -0.02 (0.11) | -0.01 (0.11) | -0.02 (0.11) | -0.02 (0.06) | -0.02 (0.06) |
| 1 | NH | Medium | 800 | 4 | 0.00 (0.09) | -0.00 (0.09) | 0.00 (0.09) | -0.00 (0.10) | -0.02 (0.05) | -0.03 (0.05) |
| 1 | NH | Medium | 800 | 7 | -0.00 (0.08) | -0.01 (0.08) | -0.00 (0.08) | -0.01 (0.08) | -0.02 (0.04) | -0.02 (0.04) |
| 2 | H | Weak | 400 | 4 | -0.06 (0.12) | -0.08 (0.12) | -0.06 (0.12) | -0.08 (0.12) | -0.03 (0.06) | -0.04 (0.06) |
| 2 | H | Weak | 400 | 7 | -0.04 (0.11) | -0.04 (0.11) | -0.04 (0.11) | -0.04 (0.11) | -0.02 (0.06) | -0.02 (0.05) |
| 2 | H | Weak | 800 | 4 | -0.04 (0.09) | -0.04 (0.09) | -0.04 (0.09) | -0.04 (0.09) | -0.03 (0.05) | -0.03 (0.04) |
| 2 | H | Weak | 800 | 7 | -0.02 (0.08) | -0.03 (0.08) | -0.02 (0.08) | -0.03 (0.08) | -0.02 (0.04) | -0.02 (0.04) |
| 2 | H | Medium | 400 | 4 | -0.04 (0.13) | -0.04 (0.12) | -0.04 (0.13) | -0.04 (0.13) | -0.04 (0.07) | -0.04 (0.06) |
| 2 | H | Medium | 400 | 7 | -0.02 (0.12) | -0.01 (0.11) | -0.02 (0.12) | -0.01 (0.11) | -0.02 (0.06) | -0.02 (0.06) |
| 2 | H | Medium | 800 | 4 | -0.00 (0.09) | -0.01 (0.09) | -0.00 (0.09) | -0.01 (0.09) | -0.03 (0.05) | -0.03 (0.05) |
| 2 | H | Medium | 800 | 7 | -0.00 (0.08) | 0.00 (0.08) | -0.00 (0.08) | 0.00 (0.08) | -0.02 (0.04) | -0.01 (0.04) |
|  |  |  |  |  |  |  |  |  |  |  |
| 2 | NH | Weak | 400 | 4 | -0.05 (0.12) | -0.06 (0.12) | -0.05 (0.12) | -0.06 (0.12) | -0.03 (0.06) | -0.03 (0.06) |
| 2 | NH | Weak | 400 | 7 | -0.03 (0.11) | -0.04 (0.11) | -0.03 (0.11) | -0.04 (0.11) | -0.01 (0.06) | -0.02 (0.05) |
| 2 | NH | Weak | 800 | 4 | -0.04 (0.09) | -0.04 (0.09) | -0.04 (0.09) | -0.04 (0.09) | -0.03 (0.04) | -0.03 (0.05) |
| 2 | NH | Weak | 800 | 7 | -0.02 (0.08) | -0.02 (0.08) | -0.02 (0.08) | -0.02 (0.08) | -0.01 (0.04) | -0.02 (0.04) |
| 2 | NH | Medium | 400 | 4 | -0.02 (0.13) | -0.02 (0.13) | -0.02 (0.13) | -0.02 (0.13) | -0.03 (0.06) | -0.03 (0.07) |
| 2 | NH | Medium | 400 | 7 | -0.02 (0.11) | -0.02 (0.11) | -0.02 (0.11) | -0.02 (0.11) | -0.02 (0.06) | -0.02 (0.05) |
| 2 | NH | Medium | 800 | 4 | 0.00 (0.10) | -0.00 (0.09) | 0.00 (0.10) | -0.01 (0.09) | -0.02 (0.05) | -0.03 (0.05) |
| 2 | NH | Medium | 800 | 7 | 0.01 (0.08) | 0.00 (0.08) | 0.00 (0.08) | 0.00 (0.08) | -0.01 (0.04) | -0.01 (0.04) |
| **Setting No. 1:** The two covariates are not correlated and they induce DIF on two distinct items  **Setting No. 2:** The two covariates are not correlated and they induce DIF on the same item **H:** Homogeneous**, NH:** Non-homogeneous | | | | | | | | | | |

**Table D1:** Bias and empirical standard error (EmpSE) associated with the estimation of $\beta_{1}$ and $\beta_{2}$ (the covariates effect on the latent variable level) under settings No. 1 and No. 2. Results are given according to the following simulation characteristics: DIF form, DIF size, sample size $n$ and number of items *J*.

**Table D2:** Bias and empirical standard error (EmpSE) associated with the estimation of $\beta_{1}$ and $\beta_{2}$ (the covariates effect on the latent variable level) under setting No. 3. Results are given according to the following simulation characteristics: DIF form, DIF size, sample size $n$ and number of items *J.*

|  |  |  |  |  | **ROSALI-DIF** | | **ROSALI-DIF  BACKWARD** | | **PCMLasso** | |
| --- | --- | --- | --- | --- | --- | --- | --- | --- | --- | --- |
|  |  |  |  |  | $\boldsymbol{\beta}_{\boldsymbol{1}}$ | $\boldsymbol{\beta}_{\boldsymbol{2}}$ | $\boldsymbol{\beta}_{\boldsymbol{1}}$ | $\boldsymbol{\beta}_{\boldsymbol{2}}$ | $\boldsymbol{\beta}_{\boldsymbol{1}}$ | $\boldsymbol{\beta}_{\boldsymbol{2}}$ |
| **Setting** | **DIF  form** | **DIF  size** | $\boldsymbol{n}$ | **J** | **Bias (EmpSE)** | **Bias (EmpSE)** | **Bias (EmpSE)** | **Bias (EmpSE)** | **Bias (EmpSE)** | **Bias (EmpSE)** |
| 3 | H | Weak | 400 | 4 | -0.14 (0.14) | -0.01 (0.13) | -0.14 (0.14) | -0.01 (0.13) | -0.08 (0.07) | -0.00 (0.07) |
| 3 | H | Weak | 400 | 7 | -0.08 (0.13) | -0.00 (0.13) | -0.08 (0.13) | -0.00 (0.13) | -0.04 (0.06) | -0.00 (0.06) |
| 3 | H | Weak | 800 | 4 | -0.13 (0.11) | -0.00 (0.11) | -0.12 (0.11) | -0.00 (0.11) | -0.08 (0.05) | -0.00 (0.05) |
| 3 | H | Weak | 800 | 7 | -0.06 (0.09) | -0.00 (0.09) | -0.06 (0.09) | -0.00 (0.09) | -0.04 (0.05) | -0.00 (0.05) |
| 3 | H | Medium | 400 | 4 | -0.19 (0.16) | 0.00 (0.14) | -0.18 (0.16) | 0.00 (0.14) | -0.14 (0.07) | 0.00 (0.07) |
| 3 | H | Medium | 400 | 7 | -0.09 (0.13) | 0.00 (0.13) | -0.09 (0.13) | 0.00 (0.13) | -0.07 (0.07) | 0.00 (0.07) |
| 3 | H | Medium | 800 | 4 | -0.05 (0.13) | 0.00 (0.10) | -0.05 (0.13) | 0.00 (0.10) | -0.13 (0.06) | 0.00 (0.05) |
| 3 | H | Medium | 800 | 7 | -0.02 (0.09) | 0.00 (0.09) | -0.02 (0.09) | 0.00 (0.09) | -0.05 (0.05) | 0.00 (0.05) |
|  |  |  |  |  |  |  |  |  |  |  |
| 3 | NH | Weak | 400 | 4 | -0.14 (0.14) | -0.00 (0.14) | -0.14 (0.14) | -0.00 (0.14) | -0.07 (0.07) | -0.00 (0.07) |
| 3 | NH | Weak | 400 | 7 | -0.06 (0.13) | -0.00 (0.13) | -0.06 (0.13) | -0.00 (0.13) | -0.03 (0.07) | -0.00 (0.06) |
| 3 | NH | Weak | 800 | 4 | -0.11 (0.10) | 0.00 (0.10) | -0.11 (0.10) | 0.00 (0.10) | -0.07 (0.05) | 0.00 (0.05) |
| 3 | NH | Weak | 800 | 7 | -0.06 (0.09) | -0.00 (0.09) | -0.06 (0.09) | -0.00 (0.09) | -0.04 (0.05) | -0.00 (0.04) |
| 3 | NH | Medium | 400 | 4 | -0.14 (0.17) | -0.00 (0.14) | -0.13 (0.17) | -0.00 (0.14) | -0.11 (0.08) | -0.00 (0.07) |
| 3 | NH | Medium | 400 | 7 | -0.06 (0.13) | 0.01 (0.13) | -0.06 (0.13) | 0.01 (0.13) | -0.05 (0.07) | 0.00 (0.07) |
| 3 | NH | Medium | 800 | 4 | -0.02 (0.12) | 0.00 (0.09) | -0.02 (0.12) | 0.00 (0.10) | -0.09 (0.06) | 0.00 (0.05) |
| 3 | NH | Medium | 800 | 7 | -0.01 (0.09) | 0.00 (0.09) | -0.01 (0.09) | 0.00 (0.09) | -0.04 (0.05) | 0.00 (0.05) |
| **Setting No. 3:** The two covariates are correlated and only one induces DIF on two items  **H:** Homogeneous**, NH:** Non-homogeneous  *Note: Highlighted cells contain a bias exceeding 0.1 in absolute value* | | | | | | | | | | |

**Table D3:** Average model standard error (ModSE) associated with the estimation of $\beta_{1}$ and $\beta_{2}$ (the covariates effect on the latent variable level). Results are given according to the following simulation characteristics: Setting, DIF form, DIF size, sample size $n$ and number of items *J.*

|  |  |  |  |  | **ROSALI-DIF** | | **ROSALI-DIF  BACKWARD** | |
| --- | --- | --- | --- | --- | --- | --- | --- | --- |
|  |  |  |  |  | $\boldsymbol{\beta}_{\boldsymbol{1}}$ | $\boldsymbol{\beta}_{\boldsymbol{2}}$ | $\boldsymbol{\beta}_{\boldsymbol{1}}$ | $\boldsymbol{\beta}_{\boldsymbol{1}}$ |
| **Setting** | **DIF  form** | **DIF  size** | $\boldsymbol{n}$ | ***J*** | **Average  ModSE** | **Average  ModSE** | **Average ModSE** | **Average  ModSE** |
| 1 & 2 | Homogeneous | Weak | 400 | 4 | 0.12 | 0.12 | 0.12 | 0.12 |
| 1 & 2 | Homogeneous | Weak | 400 | 7 | 0.11 | 0.11 | 0.11 | 0.11 |
| 1 & 2 | Homogeneous | Weak | 800 | 4 | 0.09 | 0.09 | 0.09 | 0.09 |
| 1 & 2 | Homogeneous | Weak | 800 | 7 | 0.08 | 0.08 | 0.08 | 0.08 |
| 1 & 2 | Homogeneous | Medium | 400 | 4 | 0.12 | 0.12 | 0.12 | 0.12 |
| 1 & 2 | Homogeneous | Medium | 400 | 7 | 0.11 | 0.11 | 0.11 | 0.11 |
| 1 & 2 | Homogeneous | Medium | 800 | 4 | 0.09 | 0.09 | 0.09 | 0.09 |
| 1 & 2 | Homogeneous | Medium | 800 | 7 | 0.08 | 0.08 | 0.08 | 0.08 |
| 1 & 2 | Non homogeneous | Weak | 400 | 4 | 0.12 | 0.12 | 0.12 | 0.12 |
| 1 & 2 | Non homogeneous | Weak | 400 | 7 | 0.11 | 0.11 | 0.11 | 0.11 |
| 1 & 2 | Non homogeneous | Weak | 800 | 4 | 0.09 | 0.09 | 0.09 | 0.09 |
| 1 & 2 | Non homogeneous | Weak | 800 | 7 | 0.08 | 0.08 | 0.08 | 0.08 |
| 1 & 2 | Non homogeneous | Medium | 400 | 4 | 0.13 | 0.12 | 0.13 | 0.12 |
| 1 & 2 | Non homogeneous | Medium | 400 | 7 | 0.11 | 0.11 | 0.11 | 0.11 |
| 1 & 2 | Non homogeneous | Medium | 800 | 4 | 0.09 | 0.09 | 0.09 | 0.09 |
| 1 & 2 | Non homogeneous | Medium | 800 | 7 | 0.08 | 0.08 | 0.08 | 0.08 |
| 3 | Homogeneous | Weak | 400 | 4 | 0.14 | 0.14 | 0.14 | 0.14 |
| 3 | Homogeneous | Weak | 400 | 7 | 0.13 | 0.13 | 0.13 | 0.13 |
| 3 | Homogeneous | Weak | 800 | 4 | 0.10 | 0.10 | 0.10 | 0.10 |
| 3 | Homogeneous | Weak | 800 | 7 | 0.09 | 0.09 | 0.09 | 0.09 |
| 3 | Homogeneous | Medium | 400 | 4 | 0.14 | 0.14 | 0.14 | 0.14 |
| 3 | Homogeneous | Medium | 400 | 7 | 0.13 | 0.13 | 0.13 | 0.13 |
| 3 | Homogeneous | Medium | 800 | 4 | 0.11 | 0.10 | 0.11 | 0.10 |
| 3 | Homogeneous | Medium | 800 | 7 | 0.09 | 0.09 | 0.09 | 0.09 |
| 3 | Non homogeneous | Weak | 400 | 4 | 0.14 | 0.14 | 0.14 | 0.14 |
| 3 | Non homogeneous | Weak | 400 | 7 | 0.13 | 0.13 | 0.13 | 0.13 |
| 3 | Non homogeneous | Weak | 800 | 4 | 0.10 | 0.10 | 0.10 | 0.10 |
| 3 | Non homogeneous | Weak | 800 | 7 | 0.09 | 0.09 | 0.09 | 0.09 |
| 3 | Non homogeneous | Medium | 400 | 4 | 0.15 | 0.14 | 0.15 | 0.14 |
| 3 | Non homogeneous | Medium | 400 | 7 | 0.13 | 0.13 | 0.13 | 0.13 |
| 3 | Non homogeneous | Medium | 800 | 4 | 0.11 | 0.10 | 0.11 | 0.10 |
| 3 | Non homogeneous | Medium | 800 | 7 | 0.09 | 0.09 | 0.09 | 0.09 |
| **Setting No. 1:** The two covariates are not correlated and they induce DIF on two different items  **Setting No. 2:** The two covariates are not correlated and they induce DIF on the same item  **Setting No. 3:** The two covariates are correlated and only one induces DIF on two items  *Note: As far as we know, model standard errors are not available in the GPCMLasso package. Hence these data are not available for the PCMLasso approach* | | | | | | | | |

**Appendix E:** Shiny app associated with the simulation study

*This app is interactive and allows users to:*

- *Investigate the DIF parameters estimates for ROSALI-DIF algorithms and PCMLasso*
- *Investigate bias, empirical standard error and average model standard error associated with the estimation of* $\beta_{k}$*(the effect of the covariate* $C_{k ; k=1,2}$ *on the latent variable level) for both ROSALI-DIF algorithms and PCMLasso*

**→ App available by following this** [**URL**](https://ydsphere.shinyapps.io/app_simul_result/?fbclid=IwAR0Un1sFAA5ibvqmtczgsUdN-DHXgiopVpL4Crl6NQv4C8rNGlofRhxP8TY)

**Appendix F:** Average false-positive and true-positive rates

Among the “No DIF scenarios”, we assessed the average false-positive rate (aFPR) obtained at the end of each procedure. For a single replication, the false-positive rate (FPR) is defined as the proportion of item-covariate pairs erroneously flagged for DIF among all item-covariate pairs for which no DIF was simulated. The aFPR is computed as the mean of the FPR over all 500 replications. Results are given in Table F1.

Among the “DIF scenarios”, we assessed the average false-positive rate (aFPR) and the average true-positive rate (aTPR) obtained at the end of each procedure. FPR and aFPR are defined above. Regarding the true-positive rate (TPR), it is computed for a single replication as the proportion of item-covariate pairs correctly flagged for DIF among all item-covariate pairs for which DIF was simulated. The aTPR is computed as the mean of the TPR over all 500 replications. We also considered a TPR taking into account the DIF form recovery. For a given replication:

TPR_form_ = The proportion of item-covariate pairs correctly flagged for DIF (with the right DIF form identified) among all item-covariate pairs for which DIF was simulated.

The average TPR_form_ (aTPR_form_) is computed as the mean of the TPR_form_ over all 500 replications. Results are given in Table F2.

**Table F1:** Average false-positive rate (aFPR) among scenarios with no simulated DIF computed at the end of each procedure. Results are given according to the simulation characteristics $n$(sample size), *J* (number of items) and the presence or absence of correlation between covariates $C_{1}$ and $C_{2}$

|  |  |  |  | **aFPR** |  |
| --- | --- | --- | --- | --- | --- |
| **n** | ***J*** | **Corr** | **ROSALI-DIF FORWARD** | **ROSALI-DIF BACKWARD** | **PCMLasso** |
| 400 | 4 | No | 1% | 1% | 7% |
| 400 | 4 | Yes | <1% | <1% | 7% |
| 400 | 7 | No | 1% | <1% | 4% |
| 400 | 7 | Yes | <1% | <1% | 4% |
| 800 | 4 | No | 1% | <1% | 6% |
| 800 | 4 | Yes | 1% | 1% | 6% |
| 800 | 7 | No | 1% | <1% | 4% |
| 800 | 7 | Yes | <1% | <1% | 4% |
| **Corr:** Correlation, indicates whether covariates $C_{1}$ and $C_{2}$ are correlated (=Yes) or not (=No) | | | | | |

**Table F2:** Average false-positive rate (aFPR) and average true-positive rate (aTPR and aTPR_form_) among DIF scenarios. Results are given according to the simulation characteristics: Setting, DIF form (Homogeneous H, Non-homogeneous NH), DIF size, sample size $n$, number of items $J$

| **Setting**  **No.** | **DIF Form** | **DIF size** | **n** | **J** |  | **ROSALI-DIF FORWARD** | | |  | **ROSALI-DIF BACKWARD** | | |  | **PCMLasso** | | |
| --- | --- | --- | --- | --- | --- | --- | --- | --- | --- | --- | --- | --- | --- | --- | --- | --- |
|  |  |  |  |  |  |  |  |  |  |  |  |  |  |  |  |  |
|  |  |  |  |  |  |  |  |  |  |  |  |  |  |  |  |  |
|  |  |  |  |  |  | **aFPR** | **aTPR** | **aTPR_form_** |  | **aFPR** | **aTPR** | **aTPR_form_** |  | **aFPR** | **aTPR** | **aTPR_form_** |
| 1 | H | Weak | 400 | 4 |  | 2% | 12% | 9% |  | 1% | 11% | 9% |  | 7% | 23% | 2% |
| 1 | H | Weak | 400 | 7 |  | 1% | 11% | 9% |  | 1% | 11% | 9% |  | 4% | 22% | 2% |
| 1 | H | Weak | 800 | 4 |  | 2% | 38% | 34% |  | 1% | 36% | 32% |  | 5% | 36% | 4% |
| 1 | H | Weak | 800 | 7 |  | 2% | 42% | 37% |  | 1% | 41% | 36% |  | 3% | 40% | 6% |
| 1 | H | Medium | 400 | 4 |  | 3% | 59% | 55% |  | 2% | 57% | 53% |  | 9% | 54% | 7% |
| 1 | H | Medium | 400 | 7 |  | 3% | 59% | 54% |  | 1% | 57% | 52% |  | 5% | 59% | 9% |
| 1 | H | Medium | 800 | 4 |  | 3% | 95% | 90% |  | 2% | 94% | 89% |  | 9% | 88% | 16% |
| 1 | H | Medium | 800 | 7 |  | 3% | 95% | 90% |  | 1% | 95% | 90% |  | 5% | 90% | 20% |
| 1 | NH | Weak | 400 | 4 |  | 1% | 14% | 6% |  | 1% | 14% | 5% |  | 5% | 26% | 26% |
| 1 | NH | Weak | 400 | 7 |  | 2% | 14% | 5% |  | 1% | 13% | 4% |  | 4% | 25% | 24% |
| 1 | NH | Weak | 800 | 4 |  | 3% | 45% | 15% |  | 2% | 42% | 14% |  | 5% | 47% | 46% |
| 1 | NH | Weak | 800 | 7 |  | 2% | 43% | 13% |  | 1% | 41% | 13% |  | 3% | 46% | 43% |
| 1 | NH | Medium | 400 | 4 |  | 3% | 72% | 28% |  | 1% | 70% | 27% |  | 7% | 66% | 66% |
| 1 | NH | Medium | 400 | 7 |  | 3% | 65% | 26% |  | 1% | 63% | 26% |  | 5% | 68% | 65% |
| 1 | NH | Medium | 800 | 4 |  | 3% | 98% | 60% |  | 2% | 97% | 60% |  | 9% | 93% | 93% |
| 1 | NH | Medium | 800 | 7 |  | 3% | 98% | 58% |  | 1% | 97% | 58% |  | 5% | 94% | 93% |
|  |  |  |  |  |  |  |  |  |  |  |  |  |  |  |  |  |
| 2 | H | Weak | 400 | 4 |  | 2% | 10% | 9% |  | 1% | 9% | 7% |  | 6% | 23% | 1% |
| 2 | H | Weak | 400 | 7 |  | 1% | 10% | 8% |  | 1% | 9% | 7% |  | 3% | 21% | 2% |
| 2 | H | Weak | 800 | 4 |  | 2% | 41% | 38% |  | 2% | 39% | 36% |  | 5% | 37% | 3% |
| 2 | H | Weak | 800 | 7 |  | 2% | 36% | 32% |  | 2% | 35% | 31% |  | 3% | 36% | 6% |
| 2 | H | Medium | 400 | 4 |  | 3% | 60% | 55% |  | 2% | 58% | 54% |  | 8% | 53% | 7% |
| 2 | H | Medium | 400 | 7 |  | 3% | 59% | 54% |  | 2% | 58% | 53% |  | 5% | 60% | 10% |
| 2 | H | Medium | 800 | 4 |  | 3% | 95% | 90% |  | 1% | 94% | 89% |  | 10% | 85% | 14% |
| 2 | H | Medium | 800 | 7 |  | 3% | 96% | 91% |  | 1% | 95% | 91% |  | 6% | 91% | 20% |
| 2 | NH | Weak | 400 | 4 |  | 2% | 16% | 6% |  | 1% | 15% | 6% |  | 6% | 29% | 28% |
| 2 | NH | Weak | 400 | 7 |  | 1% | 16% | 6% |  | 1% | 15% | 6% |  | 4% | 30% | 29% |
| 2 | NH | Weak | 800 | 4 |  | 2% | 50% | 15% |  | 2% | 48% | 15% |  | 4% | 46% | 44% |
| 2 | NH | Weak | 800 | 7 |  | 2% | 48% | 14% |  | 1% | 46% | 14% |  | 3% | 49% | 47% |
| 2 | NH | Medium | 400 | 4 |  | 3% | 67% | 26% |  | 2% | 64% | 26% |  | 6% | 66% | 65% |
| 2 | NH | Medium | 400 | 7 |  | 2% | 64% | 25% |  | 1% | 62% | 26% |  | 4% | 65% | 63% |
| 2 | NH | Medium | 800 | 4 |  | 3% | 98% | 56% |  | 1% | 96% | 58% |  | 7% | 92% | 91% |
| 2 | NH | Medium | 800 | 7 |  | 3% | 97% | 56% |  | 1% | 97% | 57% |  | 5% | 94% | 93% |
|  |  |  |  |  |  |  |  |  |  |  |  |  |  |  |  |  |
| 3 | H | Weak | 400 | 4 |  | 1% | 6% | 4% |  | 1% | 7% | 5% |  | 8% | 12% | <1% |
| 3 | H | Weak | 400 | 7 |  | 1% | 7% | 5% |  | 1% | 6% | 5% |  | 4% | 14% | <1% |
| 3 | H | Weak | 800 | 4 |  | 2% | 15% | 13% |  | 2% | 17% | 15% |  | 9% | 14% | 1% |
| 3 | H | Weak | 800 | 7 |  | 1% | 21% | 19% |  | 1% | 22% | 19% |  | 4% | 24% | 3% |
| 3 | H | Medium | 400 | 4 |  | 1% | 30% | 26% |  | 1% | 32% | 29% |  | 12% | 22% | 1% |
| 3 | H | Medium | 400 | 7 |  | 1% | 40% | 35% |  | 1% | 40% | 35% |  | 5% | 40% | 5% |
| 3 | H | Medium | 800 | 4 |  | 2% | 76% | 71% |  | 2% | 79% | 74% |  | 20% | 36% | 4% |
| 3 | H | Medium | 800 | 7 |  | 2% | 86% | 81% |  | 1% | 86% | 80% |  | 7% | 73% | 12% |
| 3 | NH | Weak | 400 | 4 |  | 1% | 6% | 3% |  | 2% | 7% | 3% |  | 7% | 16% | 16% |
| 3 | NH | Weak | 400 | 7 |  | 1% | 7% | 3% |  | 1% | 7% | 3% |  | 4% | 18% | 18% |
| 3 | NH | Weak | 800 | 4 |  | 2% | 23% | 8% |  | 2% | 24% | 9% |  | 8% | 23% | 22% |
| 3 | NH | Weak | 800 | 7 |  | 1% | 28% | 10% |  | 1% | 28% | 10% |  | 4% | 33% | 32% |
| 3 | NH | Medium | 400 | 4 |  | 2% | 43% | 21% |  | 2% | 47% | 22% |  | 10% | 41% | 41% |
| 3 | NH | Medium | 400 | 7 |  | 2% | 52% | 23% |  | 1% | 52% | 24% |  | 5% | 53% | 52% |
| 3 | NH | Medium | 800 | 4 |  | 3% | 90% | 53% |  | 1% | 91% | 56% |  | 15% | 72% | 72% |
| 3 | NH | Medium | 800 | 7 |  | 2% | 92% | 56% |  | 1% | 91% | 57% |  | 7% | 87% | 86% |
| **Setting No. 1:** The two covariates are not correlated and they induce DIF on two distinct items  **Setting No. 2:** The two covariates are not correlated and they induce DIF on the same item  **Setting No. 3:** The two covariates are correlated and only one induces DIF on two items | | | | | | | | | | | | | | | | |
